# Covalent Organic Framework Membranes through Sequential Imine Exchange for Precise Molecular Separation

**DOI:** 10.1007/s40820-026-02287-5

**Published:** 2026-07-30

**Authors:** Tingyuan Wang, Yanan Liu, Junhao Wu, Xiaocui Wei, Zongmei Li, Fu Zhao, Yixi Sun, Chunyang Fan, Yuhan Wang, Zhongyi Jiang

**Affiliations:** 1 School of Chemistry and Chemical Engineering, Collaborative Innovation Center of Ecological Civilization, Hainan University, Hainan, 570228 People’s Republic of China; 2 Key Laboratory for Green Chemical Technology of Ministry of Education, School of Chemical Engineering and Technology, Tianjin University, Tianjin, 300072 People’s Republic of China

**Keywords:** Covalent organic framework, Molecular separation, Dynamic imine chemistry

## Abstract

Covalent organic framework (COF) membranes with high crystallinity hold great promise in precise molecular separation, but often suffer from the intercrystalline defects and thus poor membrane-formation ability. This study reports a sequential imine exchange strategy to fabricate highly crystalline, defect-free COF membranes for precise molecular separation. Two functional amines (aromatic and aliphatic amines) are employed in highly reversible imine exchange reaction to separately conduct crystallization and defect remedy processes according to their different energy gaps. Aromatic amine, which serves as COF framework building unit, undergoes the first-step imine exchange for high crystallinity because of its lower energy gap. Afterward, the hyperbranched aliphatic amine with abundant amino groups undergoes the second-step imine exchange, affording the tight connections between adjacent crystals and the excellent membrane-formation ability. Accordingly, the COF membrane exhibits high permeance (344 L m^−2^ h^−1^ bar^−1^ for water, 462 L m^−2^ h^−1^ bar^−1^ for methanol) and rejection (> 99.9% for Congo red and Alcian blue). Meanwhile, the membrane is endowed with an ultrahigh separation factor (> 528) for mixed dye aqueous solutions and large-scale processability (> 290 cm^2^). This work offers a new strategy to fabricate highly crystalline and defect-free COF membranes, revealing their large potential in diverse practical applications.

**Supplementary Information:**

The online version contains supplementary material available at 10.1007/s40820-026-02287-5.

## Highlights

- A sequential imine exchange strategy is first proposed for the fabrication of highly crystalline and defect-free covalent organic framework (COF) membranes.
- Sequential exchange of two functional amine monomers with distinct exchange energy gaps enabled controlled crystallization and membrane formation, allowing large-area membrane fabrication (> 290 cm^2^) within 3 h.
- The fabricated COF membranes exhibited high permeance (344 L m^−2^ h^−1^ bar^−1^ for water and 462 L m^−2^ h^−1^ bar^−1^ for methanol), high rejection (> 99.9% for Congo red and Alcian blue 8GX), and an ultrahigh separation factor (> 528) for mixed dye aqueous solutions.

**Supplementary Information:**

The online version contains supplementary material available at 10.1007/s40820-026-02287-5.

## Graphical abstract

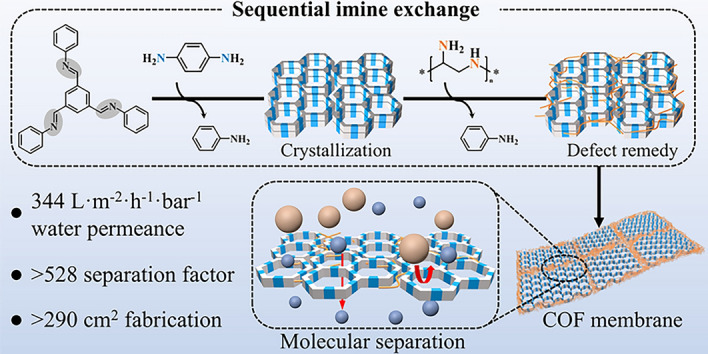

## Introduction

Membrane separation technology has provided solutions to a wide range of industrial separation challenges owing to its advantages of high efficiency and low energy consumption [1–3]. Precise and fast molecular separation is critical for resource saving and pollution control, which requires separation membranes with highly ordered structures and strict defect management [4, 5]. Covalent organic framework (COF) membranes with well-defined and ordered channels are attractive candidates for fast and precise molecule separation [6–9]. Such selective transport performance is closely associated with the high crystalline and defect-free structure of COF membranes, which enables the formation of uniform and periodic mass transport channels [10]. Imine COF membranes have been extensively studied owing to their enhanced stability, porosity, and crystallinity, and the fabrication process involves three steps: imine reaction, crystallization, and membrane formation, as clearly demonstrated by the fabrication of COF membranes using either bottom-up or top-down approaches [11–14]. However, highly crystalline COF membranes dominated by rigid framework structures often suffer from intercrystalline defects and poor membrane-formation ability due to weak interactions between crystalline domains [15]. As a commonly used imine reaction in the fabrication of imine COF membranes, imine condensation between aldehyde and amine monomers typically occurs within seconds to minutes, followed by the slow rearrangement of imine bonds to complete the crystallization [16, 17]. Owing to this temporal mismatch of imine reaction and crystallization, structural discontinuities or amorphous connections readily arise between crystalline domains, leading to the difficulty in high crystallinity and good membrane-formation ability [18, 19].

Compared to the widely used imine condensation reaction, the imine exchange reaction is another equilibrium-controlled reaction that involves substituting existing imine bonds with newly introduced amine monomers to create new imine bonds [20, 21]. Typically, monoamines initially react with an aldehyde to form imine bonds; then, the imine bond is exchanged by replacing monoamines with diamines to produce highly crystalline COF under thermodynamic driving forces [22, 23]. This reaction offers the advantage of improving the controllability over critical processes including imine reaction and crystal growth and enabling precise regulation of both growth kinetics and structural quality [24, 25], resulting in fast crystallization. Currently, the imine exchange reaction has been successfully applied to the fabrication of single-crystal COF [26, 27], substructural COF [28, 29], and highly crystalline COF powder at the minute level [30]; however, it has rarely been employed for the fabrication of COF membranes. Because highly crystalline COF membranes dominated by rigid framework structures often suffer from intercrystalline defects and poor membrane-formation ability due to weak interactions between crystalline domains [15, 31]; hence, it is crucial to separately control the crystallization and membrane-formation process. The sequential fabrication method, which decouples crystallization and membrane-formation processes and has been proven effective for constructing crystalline framework membranes including zeolite [10] and metal–organic framework (MOF) membranes [32, 33], opens an alternative avenue to constructing highly crystalline and defect-free COF membranes.

**Fig. 1:**
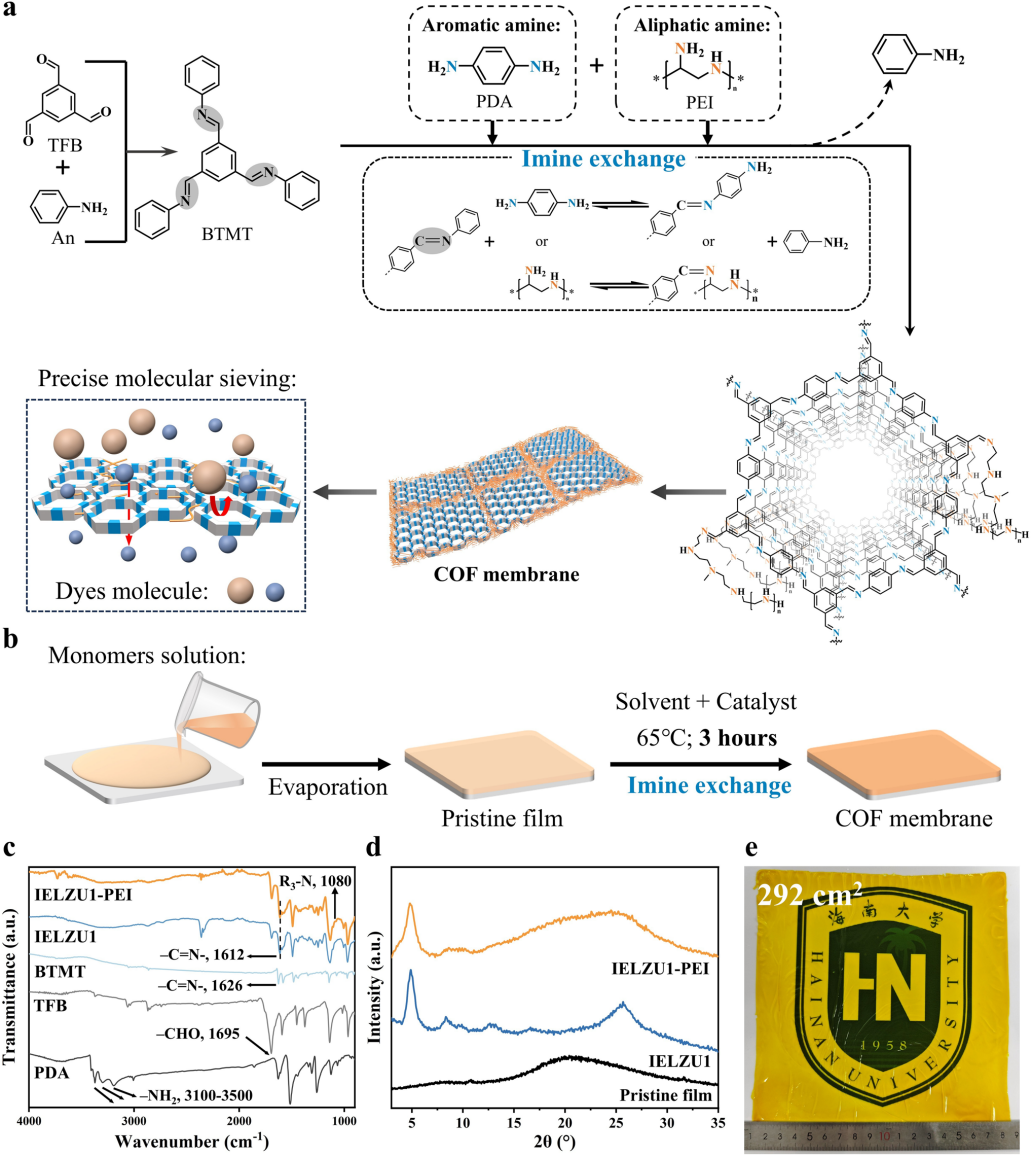
**a** Strategy illustration and application of the COF membrane. **b** Fabrication process of COF membrane via imine exchange reaction. **c** FTIR spectra of PDA, TFB, BTMT, IELZU1 membrane, and IELZU1-PEI membrane. **d** PXRD patterns of pristine film, IELZU1 membrane, and IELZU1-PEI membrane. **e** Optical image of IELZU1-PEI membrane. The IELZU1-PEI membrane was fabricated using 40 μL of PEI

Herein, we report a sequential dynamic imine exchange strategy to fabricate high crystalline, defect-free COF membranes for precise molecular separations (Fig. 1a). The N,N′,N″-(1,3,5-benzenetriyltrimethylidyne)tris (BTMT) is obtained by complete reaction of 1,3,5-triformylbenzene (TFB) with aniline (An). The resulting BTMT is dissolved and mixed with amine monomers (aromatic amine and aliphatic amine) to cast a pristine film, which then undergoes thermodynamically driven exchange of amine monomers with An to complete the imine bond exchange. The aromatic amine, serving as the COF framework building unit with a lower reaction gap (1.77 kcal mol^−1^), preferentially exchanges with An to drive the formation of the high crystalline framework. The aliphatic amine with a hyperbranched structure and abundant amino groups enables uniform dispersion of crystal seeds and promotes tight connections between adjacent crystals, undergoing the second-step imine exchange reaction with the energy gap of 4.54 kcal mol^−1^ to accomplish intercrystalline defect remedy. Three defect-free COF membranes with tunable pore sizes are fabricated within 3 h. Owing to their highly crystalline and defect-free structure, the COF membrane exhibits high separation performance and excellent large-area manufacturing ability. Our work may provide a platform for the fabrication of imine COF membranes toward diverse practical applications.

## Experimental Section

### Materials

1,3,5-Triformylbenzene (TFB, 99%) was purchased from Jilin Chinese Academy of Sciences-Yanshen Technology Co., Ltd. Benzidine (BD, 97%), N, N-dimethylacetamide (DMAC, 98%), aniline (An, 99%), sodium chloride (NaCl, 98%), and sodium sulfate (Na_2_SO_4_, 98%) were obtained from Aladdin Chemical Co., Ltd. 1,4-Phenylenediamine (PDA, 99%) was purchased from Shanghai Rhawn Chemical Technology Co., Ltd. 1,3,5-Tris(4-aminophenyl) benzene (TAPB, 97%) was supplied by Shanghai Bide Pharmaceutical Technology Co., Ltd. Polyethyleneimine (PEI, branched, Mw ≈ 600) was purchased from Shanghai D&B Biological Science and Technology Co., Ltd. Linear polyethyleneimine was purchased from Sigma-Aldrich Co., Ltd. Tetrahydrofuran (THF, 99%) was purchased from Xilong Scientific Co., Ltd. Methyl green (MG, 98%) and Phthalocyanine (Pa, 97%) were purchased from Zhengzhou Alfa Chemical Co., Ltd. Congo red (CR, 99%), Methyl orange (MO, 97%), Acid blue 25 (AB25, 97%), Eriochrome black T (EBT, 98%), Coomassie brilliant blue G-250 (CBB, 98%), and Alcian blue 8GX (AB, > 90%) were bought from Biotopped Life Science Co., Ltd. Indium tin oxide (ITO)-coated plate was supplied by Luoyang Guluo Glass Co., Ltd. All chemical reagents and solvents were used as received without further purification. Deionized water was prepared using a Milli-Q water purification system.

### Fabrication of COF Membrane

Fabrication of pristine film: BTMT (0.04 mmol) and the aromatic monomer (PDA, 0.06 mmol; TAPB, 0.04 mmol; BD, 0.06 mmol) were dissolved separately in DMAC (0.5 mL), followed by sonication for 15 min to obtain a homogeneous solution. Different amounts of PEI were added to the above homogeneous solution, and sonication was continued for 15 min to obtain solutions with different PEI additions. The resulting solution was cast uniformly onto an indium tin oxide-coated plate (area: approximately 10.2 cm^2^), and a pristine film was obtained by evaporating the solvent in an oven (60 °C). Fabrication of COF membrane from pristine film: The pristine film on the indium tin oxide-coated plate was placed in a mixture of solvent and catalyst at a defined ratio to undergo the imine exchange reaction. After the reaction, the membrane was easily detached from the plate by placing it in deionized water, resulting in a freestanding COF membrane, which was subsequently washed by soaking in ethanol.

### Characterization

Powder X-ray diffraction (PXRD) data were collected on a Rigaku Smart Lab equipment in the range of 3°–35°. The membranes were etched and mounted on the PXRD holder for characterization. TEM (Thermo Scientific Talos F200X G2) was employed to acquire high-resolution pattern of samples. The membrane was embedded in resin, sectioned (Leica EM UC7 + FC7), and then drop-cast onto an ultrathin carbon film (BZ11032a, Beijing Zhongjing Keyi Technology Co., Ltd.) supported on a lacey Formvar/carbon film TEM grid. The Fourier transform infrared spectroscopy (FTIR) spectra were recorded over a range of 500–4000 cm^−1^ on a Bruker INVENIO-S (equipped with a universal attenuated total reflection). SEM images were recorded by field emission scanning electron microscope (Thermo Scientific Verios G4 UC). N_2_ adsorption/desorption isotherms were recorded on Quantachrome Autosorb IQ MP gas adsorption analyzers at 77 K using a liquid nitrogen bath. Several membranes were first fabricated and then ground for porosity evaluation. All the membrane samples were degassed at 120 °C for 6 h under vacuum before N_2_ analysis. Tensile tests were conducted using an electronic universal testing machine (WD-50 N, Jilin Guanteng Automation Technology Co., Ltd. China) at a stretching rate of 50 mm min^−1^ to evaluate the mechanical properties of the membranes.

### Separation Performance Measurements

A laboratory-made dead-end filtration cell, equipped with magnetic stirrer and nitrogen flow for pressure maintenance, was used for molecular transport. Before measurement, the COF membranes were subjected to initial pressure of 1.5 bar for 0.5 h to obtain the steady flux. Permeance was measured at driving pressure at 1.0 bar. The mass of permeate solution was recorded by a balance automatically per minute, and the permeance (*P*, L m^−2^ h^−1^ bar^−1^) was calculated as follows:

1 $$\begin{array}{*{20}c} {P = \frac{V}{A\Delta t\Delta P} } \\ \end{array}$$

where *V* (L) is the volume of permeate; *A* (m^2^) is the effective membrane area; *∆t* (h) is the permeating time; *∆P* is the driving pressure. The filtration and separation tests were demonstrated by separating dyes in molecular weight range 100–1300 Da with different charges and catenulate. The permeate solution was collected after 0.2 h, and the dyes rejection (%) was calculated as follows:

2 $$\begin{array}{*{20}c} {R = \frac{{C_{{\mathrm{f}}} - C_{p} }}{{C_{{\mathrm{f}}} }} \times 100\% } \\ \end{array}$$

where *C*_*p*_ and *C*_f_ (ppm) are the concentrations of filtration and feed solutions, respectively. The mixed dye aqueous solutions were obtained by mixing the single-component solutions with the same volume. Because the pristine single-component solution had the same molarity (100 ppm), each component in the resulting mixtures had the same mole percent of 50%. The other testing conditions identify with the single-component separation. According to the Van’t Hoff equation, the osmotic pressure (around 0.0025 bar) originating from the feed solution was far less than the operation pressure (1.0 bar). The total solution permeance and the rejection rates of each component for the mixture separation still can be calculated by Eqs. (1) and (2), respectively. The separation factor (SF) of the mixture in the permeate side was calculated by Eq. (3):

3 $$\begin{array}{*{20}c} {SF=\frac{Cp1}{Cf1}/\frac{Cp2}{Cf2}=\frac{Cp1}{Cp2}=\frac{1-R1}{1-R2}\times 100\%} \\ \end{array}$$

Deionized water was used for water transport, while reagent grade organic solvents were used for organic solvent nanofiltration. All dyes were dissolved in water for molecular separation. To make sure that the COF membranes did not act as adsorbent, the first 10 mL permeate of each test was discarded and then the concentration in the permeate was calculated at different intervals (10 mL each for 10 cycles). Similarly, the permeance was also recorded at different pressures ranging from 0.5 to 3.0 bar. Long-term water permeance was obtained by recording data at various intervals at 1.0 bar. Similarly, the performance was evaluated at various cycles of operation. After each cycle, the membrane was washed with water before next cycle.

## Results and Discussion

### Fabrication and Characterization of COF Membranes

COF-LZU1 was selected as our model COF, which is a prototypical imine COF condensed from TFB and PDA [34]. BTMT was synthesized by fully reacting TFB with An (Fig. S1), because An was selected as the monoamine due to its reactivity being comparable to that of PDA. As shown in Figs. 1b and S2, the BTMT and amine monomers were dissolved in N,N-dimethylacetamide and cast into a liquid film, and the solvent was evaporated to form a pristine film. Then, a sequential imine exchange reaction was carried out in the presence of solvent and catalyst to obtain COF membranes (the membrane fabricated from BTMT and PDA is denoted as IELZU1, while the membrane fabricated from BTMT, PDA, and PEI is denoted as IELZU1-PEI). Specifically, An is replaced by aromatic and aliphatic amines under thermodynamic driving forces to accomplish the imine bond exchange. We first investigated the effect of the ratio of solvent to catalyst for imine exchange reaction. As shown in Fig. S3, the IELZU1 membrane exhibited optimal crystallinity when the ratio of solvent to catalyst was 6:4. Furthermore, we explored the influence of different solvents on the crystallinity of the resulting COF membranes. As shown in Fig. S4, the IELZU1 membrane showed better crystallinity when THF with a low boiling point was used as the solvent. More importantly, the low boiling point of THF allowed us to conduct the imine exchange reaction at a lower temperature and also facilitated solvent removal after membrane fabrication. Subsequently, the effect of reaction temperature on membrane crystallization was investigated in the range of 20–80 °C. As shown in Fig. S5, the IELZU1 membrane fabricated at 65 °C displayed the optimal crystallinity. When the reaction temperature was further increased, the crystallinity of membrane decreased. This reduction might result from the use of tetrahydrofuran (boiling point of tetrahydrofuran: 66 °C under ambient pressure) as solvent, which boils at higher temperatures, leading to an unstable reaction environment and consequently hindering the ordered growth of COF membrane.

FTIR spectra showed the stretching vibration peak at 1626 cm^−1^ attributed to the C=N bond, indicating the formation of imine bonds in BTMT (Fig. 1c). Meanwhile, the N–H bands of amine in the 3100–3500 cm^−1^ region and the aldehyde band at 1695 cm^−1^ were absent, confirming the absence of unreacted amino or aldehyde groups in BTMT. As shown in Fig. 1d, after 3 h imine exchange reaction, the pristine film was reconstructed into a highly crystalline COF membrane, and the addition of an appropriate amount of PEI did not significantly impact crystallinity. The FTIR spectrum of IELZU1 and IELZU1-PEI showed that the stretching band of the –C=N– group shifted from 1626 cm^−1^ (in BTMT) to 1612 cm^−1^, indicating the progress of imine bond exchange, which is consistent with previously reported findings [35]. Furthermore, the appearance of R_3_–N stretching vibration at 1080 cm^−1^ in IELZU1-PEI, attributed to the tertiary amine band (Fig. S6) [36], along with supporting evidence from solid-state ^13^C NMR spectra, confirmed the incorporation of PEI (Fig. S7) [37]. Meanwhile, scanning electron microscopy (SEM) revealed a marked morphological change between the surface of the pristine film and that of COF membrane (Figs. S8–S10). Extending the imine exchange reaction time, the crystallinity of the IELZU1-PEI membrane showed no obvious change, indicating that the reaction had been completed within 3 h (Fig. S11). Thanks to the simple fabrication method and the rapid crystallization process, IELZU1-PEI membranes with a maximum area of more than 290 cm^2^ were fabricated within 3 h (Figs. 1e and S12).

### Elucidating the Formation Mechanism of IELZU1-PEI Membranes

**Fig. 2:**
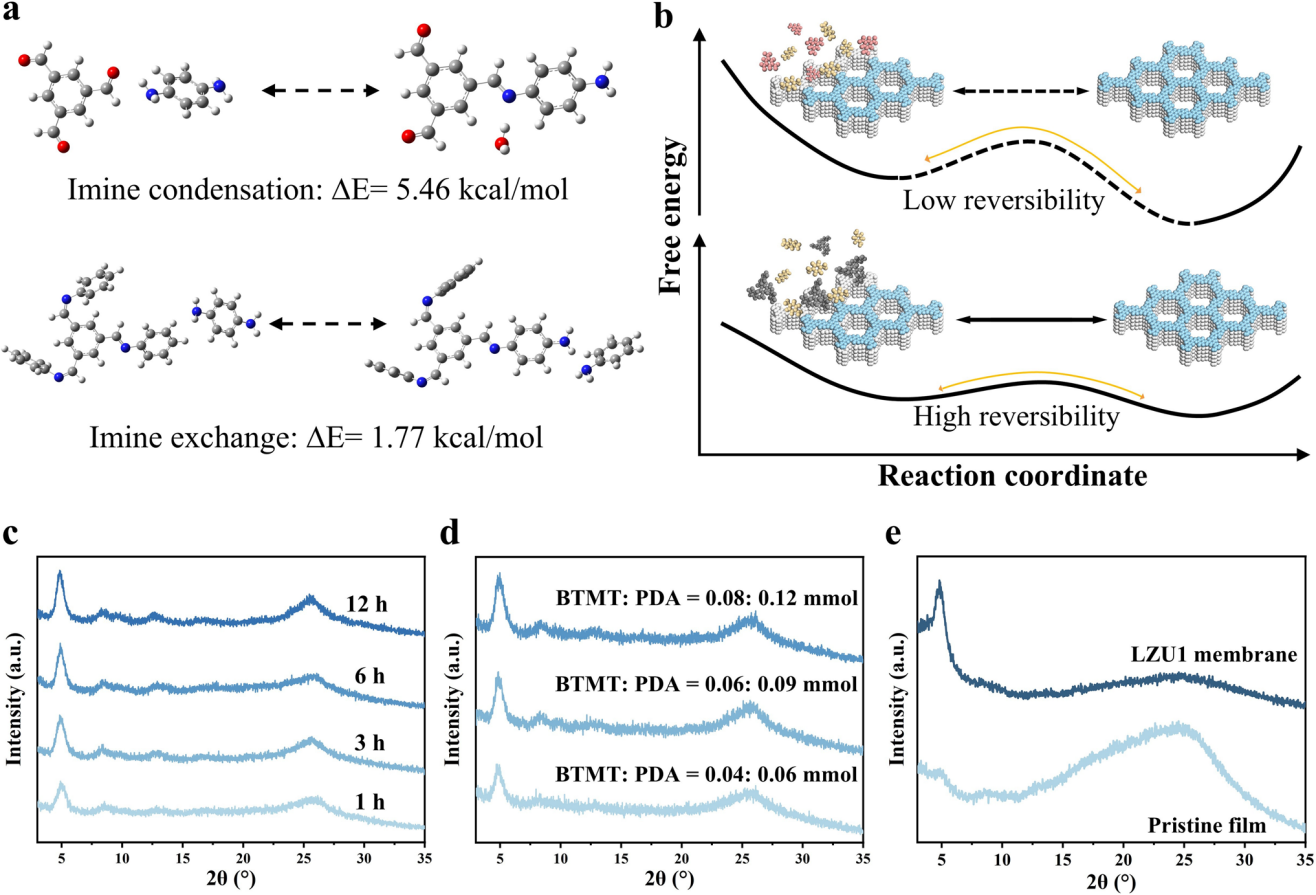
**a** Scheme showing the computed total energy gap between the reactant and product. **b** Reversibility of two reactions. The upper curve corresponds to the imine condensation reaction, and the lower curve corresponds to the imine exchange reaction. **c** PXRD patterns of IELZU1 membranes under different imine exchange times. **d** PXRD patterns of IELZU1 membranes fabricated by casting solutions with different concentrations. **e** PXRD patterns of LZU1 membrane fabricated via imine condensation reaction in the presence of An

The introduction of the aromatic imine exchange reaction enhanced the reversibility of the formation and decomposition of imine bonds during the fabrication of IELZU1 membranes. Density functional theory (DFT) calculations were used to elucidate this exchange protocol in more detail. The total energy gap between the reactants and products indicated the difference in reversibility between the two reactions (Figs. 2a and S13, S14), and the higher reversibility of imine exchange reaction for aromatic amine prevented the system from entering kinetic traps during imine condensation reaction [25, 38]. In contrast to the imine condensation reaction, which proceeded through hydrolysis and recondensation to achieve imine bond cleavage and reorganization for advancing crystallization, the imine exchange reaction accomplished this solely via aromatic amine substitution [39]. Most importantly, the strong nucleophilicity of aromatic amine (compared to water) enabled rapid imine bond exchange, thereby guaranteeing efficient self-healing of COF framework [40]. The reaction progress provided by imine exchange reaction ensured a highly efficient and high-quality crystallization process (Fig. 2b). As shown in Fig. 2c, the IELZU1 membrane displays initial crystallinity after 1 h of aromatic imine exchange reaction and reaches a highly crystalline structure at 3 h. However, the IELZU1 membrane exhibited imperfections and fractures after undergoing imine exchange reaction at low monomer concentrations (Fig. S15a). This was attributed to the diffusion of An into the solution during the imine exchange reaction in the presence of solvent and catalyst, while PDA and adjacent BTMT formed the framework. This spatial rearrangement led to intercrystalline defects, and similar phenomena were also reported in previous literature [41]. Increasing the concentration of PDA in the casting solution improved crystallinity; however, it also caused more defects, resulting in a powder-like product after the imine exchange reaction (Figs. S16 and S17), further confirming the above viewpoint. Increasing the concentrations of both BTMT and PDA in the casting solution produced a more continuous pristine film with a denser packing of reactive monomers, which promoted sufficient contact and exchange during the imine exchange process and thus resulted in macroscopically intact membranes with greater thickness and significantly enhanced crystallinity (Figs. 2d and S15b, c). However, the increase in the membrane thickness did not eliminate the generation of defects during the imine exchange reaction, even when the crystallinity was improved (Figs. S18 and S19). This indicated that intercrystalline defects originated from the spatial rearrangement of monomers during the imine exchange reaction; thus, increasing the membrane thickness could not compensate for these defects.

As a comparative experiment, we fabricated LZU1 membranes via the imine condensation reaction. We fabricated the pristine film using TFB and PDA as monomers and then subjected it to the same treatment process used for the fabrication of the COF membrane via imine exchange reaction. As shown in Figs. S20 and S21, the pristine film completely fractured during this process, and the PXRD pattern confirmed that the resulting product was amorphous. In contrast, the pristine film fabricated from TFB, PDA, and An could be reconstructed into a crystalline COF membrane after the same treatment process used for the fabrication of the COF membrane via imine exchange reaction (Fig. 2e). However, the pristine film fabricated in the presence of An exhibited film fracture. On the one hand, the introduction of An reduced the crosslinking degree of the pristine film. On the other hand, the pristine film also exhibited crystallinity, which may cause the crystalline and amorphous regions to respond differently to the stress generated during solvent evaporation, thereby leading to stress concentration and ultimately membrane fracture (Fig. S22). The comparative experiments confirmed the necessity of employing BTMT for the fabrication of IELZU1 membranes. The imine exchange reaction via aromatic amine enabled rapid crystallization under mild conditions, but poor membrane-formation ability and numerous defects still hindered the application in nanofiltration.

**Fig. 3:**
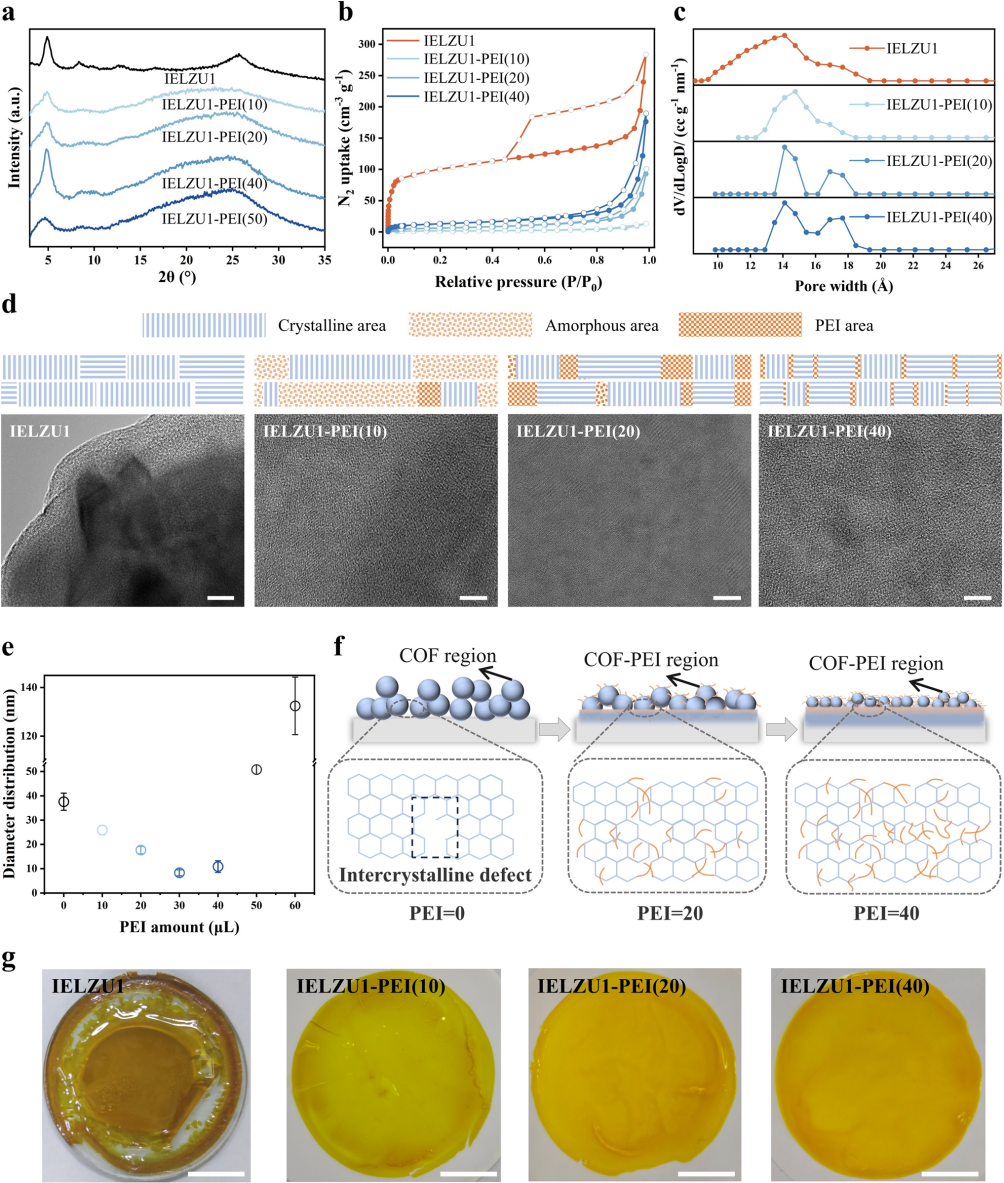
**a** PXRD patterns, **b** N_2_ adsorption–desorption isotherms, **c** pore size distribution, and **d** TEM images of IELZU1 and IELZU1-PEI(x) membranes. Pore size distribution was calculated using nonlocal density functional theory (NLDFT), and the scale bar in TEM images was 10 nm. **e** DLS profile of IELZU1 and IELZU1-PEI(x) casting solution. **f** Schematic images of IELZU1 and IELZU1-PEI(x) membranes. **g** Optical images of IELZU1 and IELZU1-PEI(x) membranes (scale bar: 1 cm)

Figure 3a shows the effect of different PEI amounts on the crystallinity of the IELZU1-PEI membrane after 3 h of imine exchange reaction. It was clearly observed that with the increase in PEI amount, the crystallinity of IELZU1-PEI membrane first increased and then decreased. The addition of an appropriate amount of PEI improved the membrane-formation ability while maintaining crystallinity. Further calculations reveal that the imine exchange of the aliphatic amine requires a higher energy gap (1.77 kcal mol^−1^ for PDA and 4.54 kcal mol^−1^ for PEI), thereby guaranteeing the imine exchange of PDA and PEI (Figs. S23 and S24) [36]. Compared with the imine exchange of PDA alone, the sequential imine exchange of PDA and PEI proceeds over a longer timescale while maintaining the crystallinity of the resulting framework. The N_2_ sorption isotherm was tested on the COF membrane at 77 K. Compared with IELZU1 membrane, the incorporation of PEI led to a reduction in Brunauer–Emmett–Teller (BET)-specific surface areas in IELZU1-PEI membranes, which was mainly attributed to the formation of amorphous crosslinked PEI-rich regions with relatively low surface area (Fig. 3b). For the IELZU1-PEI membranes, when the PEI amount increased from 10 to 40 μL, the membrane crystallinity gradually increased, and the BET surface area also slightly increases, suggesting that the improvement in framework ordering and pore regularity. However, when the PEI amount reached 50 μL, both the crystallinity and the BET surface area decreased significantly, indicating that excessive PEI not only strongly disrupted the ordered reconstruction but also occupied the pores of the COF framework, leading to fewer accessible ordered pores and more disordered polymer-rich regions (Figs. S25 and S26). These results suggested that an appropriate amount of PEI promoted the formation of crystalline regions and facilitated uniform PEI dispersion. The pore size distribution of both IELZU1 and IELZU1-PEI(10) membranes was centered at 14 Å (Fig. 3c), which was highly consistent with the pore size distribution of IELZU1 powder (synthesized through imine exchange reaction, denoted as IELZU1 powder) (Figs. S27 and S28) [34, 42]. Interestingly, both IELZU1-PEI(20) and IELZU1-PEI(40) membranes showed pore size distributions centered at 14 and 17 Å. We believed that the emergence of the pore size distribution at 17 Å was attributed to the free volume generated by the uniform distribution of PEI within IELZU1-PEI membranes. Furthermore, the COF membrane fabricated using linear PEI exhibited obvious inhomogeneity and fracture (Fig. S29), demonstrating the importance of hyperbranched PEI for the interconnection of adjacent COF crystals.

The crystal structure of IELZU1 and IELZU1-PEI membranes was further observed by transmission electron microscopy (TEM). As shown in Fig. 3d, IELZU1 membrane showed a clearly ordered lattice over an area of tens or even hundreds of square nanometers. As PEI was added, the IELZU1-PEI(x) membranes showed the coexistence of non-crystalline and crystalline regions. As the PEI amount increased (PEI = 10, 20, and 40 μL), the proportion of crystalline regions in the IELZU1-PEI(x) membranes gradually grew, which corresponded to the changes in the overall crystallinity of membranes. An excess of PEI led to a decrease in the crystallinity of IELZU1-PEI(50) membrane, and its TEM image exhibited a clearly amorphous structure (Fig. S30). The magnified TEM images clearly showed the ordered lattice of IELZU1-PEI membranes. In addition, we observed that the interplanar spacing of the (001) plane of COF membranes changed with different amount of added PEI, which further indicated that PEI participated in the growth process of COF crystals (Figs. S31–S34).

Further investigations of the impact of PEI on IELZU1-PEI membrane fabrication were conducted using dynamic light scattering (DLS). As shown in Figs. 3e and S35, the addition of an appropriate amount of PEI facilitated the uniform dispersion of crystal seeds, thereby ensuring the uniform and dense structure of both the pristine film and the COF membrane. Meanwhile, PEI with a hyperbranched structure contributed to improving the connectivity of crystals in the COF membrane, thereby achieving the fabrication of a defect-free membrane (Figs. 3f–g and S36). The introduction of PEI helped connect adjacent COF crystallites and remedy intercrystalline defects through its abundant amine groups and flexible polymer chains, thereby improving membrane continuity and reducing stress concentration. As a result, the mechanical properties of the COF membranes gradually improved as the PEI amount increased from 10 to 40 μL, with IELZU1-PEI (40) showing the best mechanical performance (tensile strength: 18.9 MPa; elongation at break: 56.2%) (Fig. S37). It was worth noting that the top and bottom surfaces of IELZU1-PEI(x) membrane exhibited different morphologies (Figs. S38 and S39). This phenomenon was attributed to the varying environments experienced by the membrane surfaces during the transformation from the pristine film to the COF membrane—the top surface contacting with solvent and catalyst, and the bottom surface contacting with indium tin oxide-coated plate—which led to different degrees of imine exchange reaction. The interface between the loose and dense layers could be observed in the cross-sectional SEM images (Fig. S40).

### Nanofiltration Performance

**Fig. 4:**
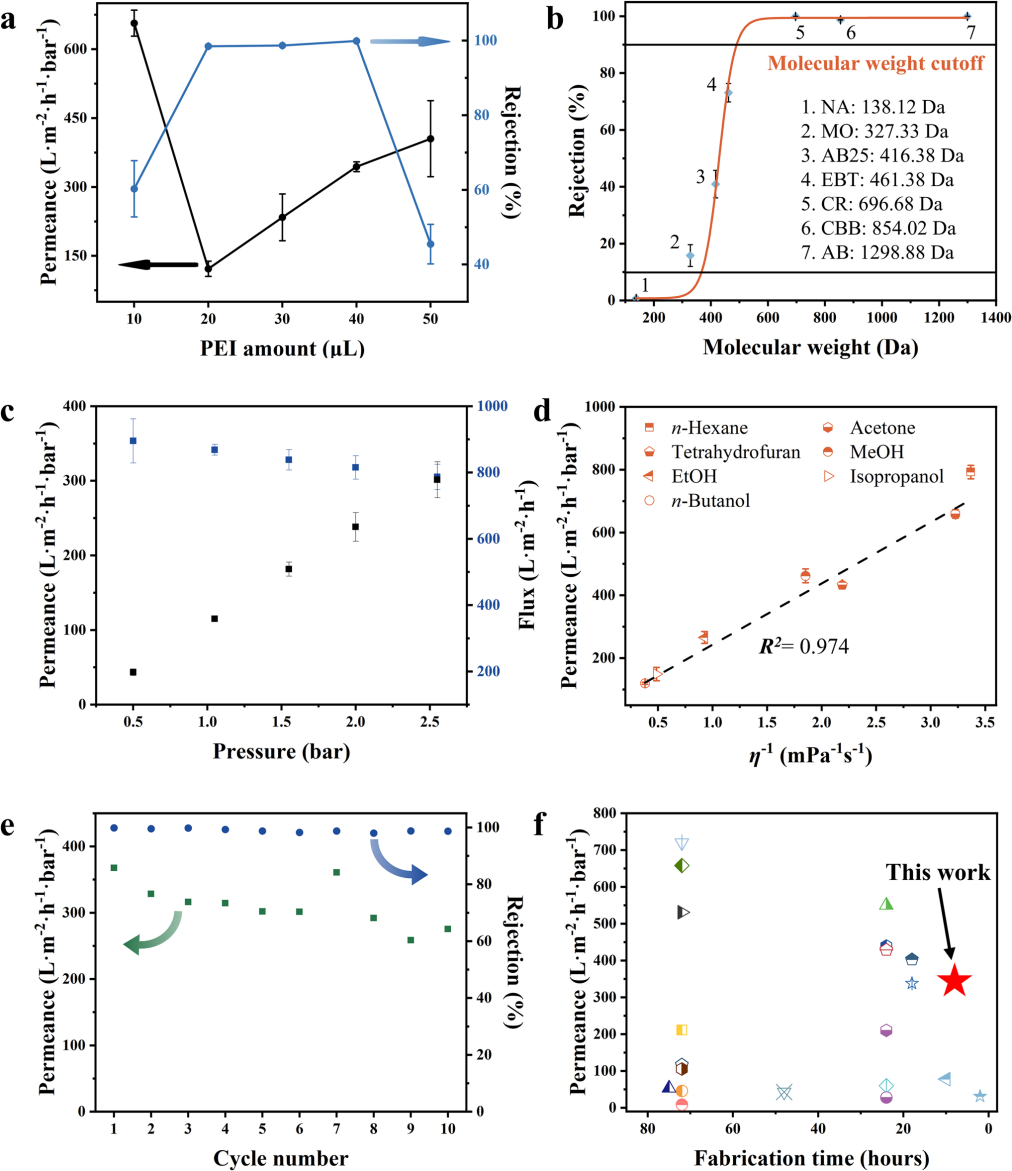
**a** Permeance and CR rejection of IELZU1-PEI membrane with different PEI amounts. **b** Rejection of dyes with different molecular weights of the IELZU1-PEI membrane. The rejection curve was fitted using the DoseResp model. **c** Variation of the flux and permeance of IELZU1-PEI membrane under varying pressure. **d** Permeance for organic solvents of IELZU1-PEI membrane with a function of their inverse viscosity. **e** Cycle performance for CR rejection of IELZU1-PEI membrane. **f** Comparison of the permeance and fabrication time of IELZU1-PEI membrane with the most recently reported COF membranes. Note that in this figure, except for **a**, the PEI amount used in all other figures was 40 μL. Dye solution concentration: 100 ppm; operation pressure: 1 bar

The nanofiltration performance of IELZU1-PEI membrane was evaluated using the laboratory-made dead-end filtration setup and dye aqueous solution. As shown in Fig. 4a, with the increase of PEI amount, the Congo red (CR) rejection of IELZU1-PEI membranes first increased and then decreased, while the corresponding membrane permeance first decreased and then increased. When the PEI amount was 40 μL, the IELZU1-PEI membrane exhibited optimal nanofiltration performance, with water permeance of 344 ± 10 L m^−2^ h^−1^ bar^−1^ and CR rejection rate exceeding 99.9%. However, excessive PEI (50 μL) disrupted COF framework reconstruction during imine exchange, forming disordered flexible regions that weakened crystalline-channel-dominated sieving and reduced rejection performance. We investigated the rejection effect of IELZU1-PEI on a series of dye solutes with different molecular weight. As shown in Figs. 4b and S41, the molecules with molecular weight > 490 Da (CR, Mw = 696.68; Coomassie brilliant blue (CBB), Mw = 854.02; Alcian Blue 8GX (AB), Mw = 1298.88) could be effectively rejected by IELZU1-PEI, with rejection over 90%.

To further eliminate the influence of adsorption on dye separation, the IELZU1-PEI membrane was immersed in dye solutions with different charges for 24 h to ensure adsorption saturation before the separation tests. As shown in Fig. S42, the membrane after saturation adsorption did not exhibit a decrease in rejection, which indicated that the rejection of dyes was based on size exclusion rather than adsorption. The Zeta potential of the membrane surface further indicated that the Donnan exclusion effect made a minor contribution to the dye rejection (Fig. S43). The linear increase in water flux of membranes with the increase in transmembrane pressure indicated the robustness of COF framework (Fig. 4c). The transport characteristics of various organic solvents through the COF membrane were tested. As shown in Fig. 4d, there was a linear relation between the permeance of different solvents through the IELZU1-PEI membrane and the inverse of their viscosity (*η*^−1^). The most commonly used model solvent, methanol, with a viscosity of 5.4 × 10^–4^ Pa s, showed a high permeance of 462 ± 22 L m^−2^ h^−1^ bar^−1^.

Cyclic performance was a key factor in advancing the practical application of COF membranes. The IELZU1-PEI membrane demonstrated impressive dye rejection efficiency even after 10 cycles (Fig. 4e). Moreover, it maintained high rejection for dye solutions of varying concentrations (20–100 ppm) (Fig. S44). Additionally, after continuous 16 h nanofiltration testing, there was no significant change in dye rejection (Fig. S45). As shown in Fig. 4f and Table S1, the imine exchange reaction greatly reduced the fabrication requirements of the COF membranes, including the fabrication time and temperature, while the IELZU1-PEI membrane also exhibited performance competitive with that of state-of-the-art nanofiltration COF membranes. Furthermore, as shown in Fig. S46 and Table S2, the performance comparison with other COF membranes based on LZU1 further demonstrated the advantages of the imine exchange reaction.

**Fig. 5:**
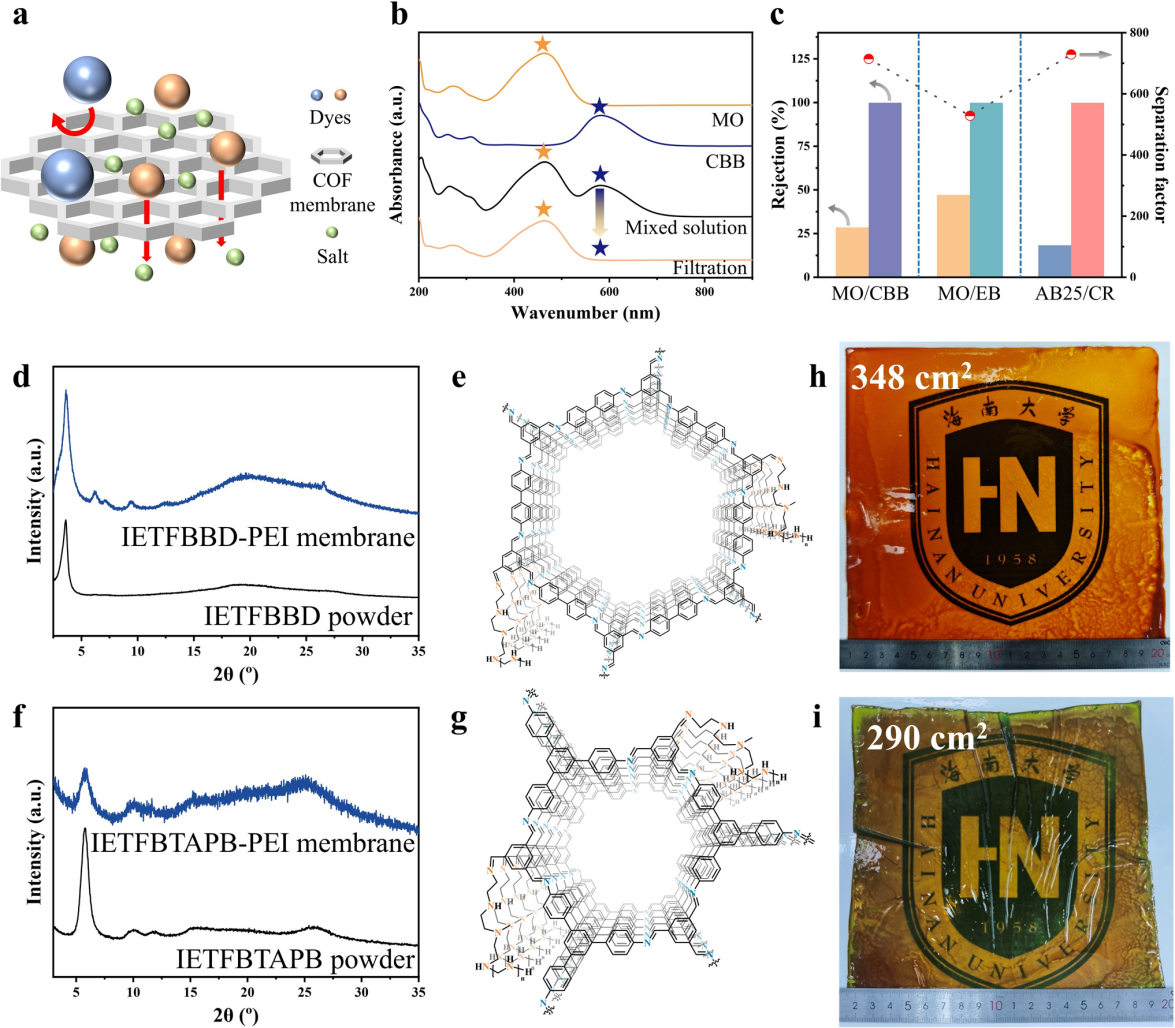
**a** Schematic illustration of the separation mechanism. **b** UV–Vis spectra of the selective separation of the MO-CBB mixed solution. **c** Molecular separation performance of IELZU1-PEI membrane. **d** PXRD patterns of IETFBBD powder with IETFBBD-PEI membrane. **e** Structure of the IETFBBD-PEI membrane. **f** PXRD patterns of IETFBTAPB powder with IETFBTAPB-PEI membrane. **g** Structure of the IETFBTAPB-PEI membrane. **h** Optical images of IETFBBD-PEI membrane and **i** IETFBTAPB-PEI membrane. The COF membranes shown were fabricated using 40 μL of PEI

To explore the molecular separation performance of IELZU1-PEI membrane, we attempted to separate mixed dye aqueous solutions containing probe molecules with different sizes. The mixed dye separation experiment included three systems: Methyl orange (MO)-CBB, MO-Evans blue (EB), and Acid blue 25 (AB25)-CR (Fig. 5a, b). Surprisingly, the separation factors of these three systems reached 714 (for MO-CBB), 528 (for MO-EBT), and 729 (for AB25-CR) (Figs. 5c and S47). Thanks to its defect-free and highly crystalline structure, the IELZU1-PEI membrane demonstrated excellent molecular separation performance for mixed dye aqueous solutions.

### Broad Applicability of Sequential Imine Exchange Strategy

To explore the broad applicability of the sequential imine exchange strategy, we fabricated COF membranes with different pore sizes. As shown in Fig. 5d, e, the IETFBBD-PEI membrane exhibited a sharp diffraction peak at 2*θ* = 3.61°, corresponding to the reflection of the (100) plane, while the diffraction peaks at 2*θ* = 6.25° and 7.01° were attributed to the (110) and (200) planes, respectively, indicating that the IETFBBD-PEI membrane possessed high crystallinity [43]. Similarly, IETFBTAPB-PEI showed a diffraction peak at 2*θ* = 5.87°, corresponding to the (100) plane, while weaker diffraction peaks appearing at 2*θ* = 9.97° and 11.75° corresponded to the (110) and (220) planes, respectively, confirming the formation of crystalline COF membrane (Fig. 5f, g) [44]. As shown in Fig. S48, further tests were conducted on the nanofiltration performance of two COF membranes. Interestingly, we observed that the water permeance of COF membranes was directly related to the theoretical pore size of the COF framework. The IETFBBD-PEI membrane, with the largest pore size (theoretical pore size: 24 Å), achieved water permeance of 600 ± 51 L m^−2^ h^−1^ bar^−1^, while the IETFBTAPB-PEI membrane, with the smallest pore size (theoretical pore size: 14 Å), had water permeance of 44 ± 1 L m^−2^ h^−1^ bar^−1^. This indicated that the permeability of COF membranes was directly related to the rigid pore structure of COF. Thanks to the rapid crystallization of the imine exchange reaction and the simplicity of the solution casting method, we were able to scale up the COF membrane to 290 cm^2^ under mild conditions (Fig. 5h, i).

## Conclusions

In summary, we have fabricated highly crystalline, defect-free COF membranes through a sequential imine exchange strategy. The crystallization and defect remedy are separately controlled by the aromatic and aliphatic amines owing to their different energy gap for the imine exchange reaction. The aromatic amine drives reversible imine exchange for rapid framework crystallization, while the hyperbranched aliphatic amine improves crystal connectivity and remedies intercrystalline defects for membrane formation. The resulting membrane exhibits high permeance (344 L m^−2^ h^−1^ bar^−1^ for water and 462 L m^−2^ h^−1^ bar^−1^ for methanol) and exceptional rejection performance (99.9% for CR, 98.7% for CBB, and 99.9% for AB). Moreover, we have demonstrated the generality of this strategy by fabricating a series of COF membranes with pore sizes spanning 14–24 Å and membrane area exceeding 290 cm^2^, which exhibit separation factor > 528 for mixed dye aqueous solutions. Our work provides an innovative and versatile platform for the fabrication of large-area, defect-free COF membranes with well-defined structures under mild conditions, representing a significant step toward their practical applications in advanced molecular separation processes.

## Supplementary Information

Below is the link to the electronic supplementary material.

Supplementary file1 (DOCX 16311 KB)
