# Supplementary material for: Covalent Organic Framework Membranes through Sequential Imine Exchange for Precise Molecular Separation
Source: Nanomicro Lett. 2026 Jul 30;19:15. doi: 10.1007/s40820-026-02287-5 (PMC13424015; doi:10.1007/s40820-026-02287-5)
Supplement: Supplementary file 1 — Supplementary file1 (DOCX 16311 KB) [file 40820_2026_2287_MOESM1_ESM.docx]

Supporting Information for

**Covalent Organic Framework Membranes through Sequential Imine Exchange for Precise Molecular Separation**

Tingyuan Wang^1^, Yanan Liu^1,^ *, Junhao Wu^1^, Xiaocui Wei^1^, Zongmei Li^1^, Fu Zhao^1^, Yixi Sun^1^, Chunyang Fan^1^, Yuhan Wang^1^ and Zhongyi Jiang^1,^ ^2,^ *

^1^ China Chemistry and Chemical Engineering, Collaborative Innovation Center of Ecological Civilization, Hainan University, Hainan 570228, P. R. China

^2^ Key Laboratory for Green Chemical Technology of Ministry of Education, School of Chemical Engineering and Technology, Tianjin University, Tianjin 300072, P. R. China

* Corresponding authors. E-mail: [zhyjiang@tju.edu.cn](mailto:zhyjiang@tju.edu.cn) (Zhongyi Jiang); [liuyanan@hainanu.edu.cn](mailto:liuyanan@hainanu.edu.cn) (Yanan Liu)

**S1 Supplementary Text**

***S1.1 Synthesis of BTMT***

BTMT was synthesized according to the literature [S1, S2]. A mixture of TFB (1.8 mmol) and aniline (8.6 mmol) was dissolved in anhydrous ethanol (10 mL) within a 50 mL flask. The solution was heated and stirred for 24 hours at 80℃ under nitrogen protection. After washing with ethanol three times, the solid products were collected and dried to afford the product as a white solid.

***S1.2 Synthesis of IELZU1 powder***

A mixture of BTMT (0.4 mmol) and PDA (1.8 mmol) was dissolved in THF (15 mL) in a 50 mL flask. Then, aqueous acetic acid (10 mL, 12 M) was added, and the solution was refluxed with vigorous stirring for 3 hours at 65℃. The products were collected by filtration, washed thoroughly using a Soxhlet extractor, and then dried under vacuum at 120℃ for 12 hours to obtain IELZU1 powder.

***S1.3 Synthesis of IETFBTAPB powder***

A mixture of BTMT (0.4 mmol) and TAPB (0.4 mmol) was dissolved in THF (10 mL) within a 25 mL flask. Then, aqueous acetic acid (4 mL, 12 M) was added, and the solution was refluxed with vigorous stirring for 3 hours at 65℃. The products were collected by filtration, washed thoroughly using a Soxhlet extractor, and then dried under vacuum at 120℃ for 12 hours to obtain IETFBTAPB powder.

***S1.4 Synthesis of IETFBBD powder***

A mixture of BTMT (0.4 mmol) and BD (0.6 mmol) was dissolved in THF (10 mL) within a 25 mL flask. Then, aqueous acetic acid (4 mL, 12 M) was added, and the solution was refluxed with vigorous stirring for 3 hours at 65℃. The products were collected by filtration, washed thoroughly using a Soxhlet extractor and then dried under vacuum at 120℃ for 12 hours to obtain IETFBBD powder.

***S1.5 Fabrication of LZU1 membrane via imine condensation reaction***

The LZU1 membrane prepared by imine condensation follows a similar process to the IELZU1 membrane. Fabrication of pristine film: TFB (0.04 mmol) and PDA (0.06 mmol) were dissolved separately in DMAC (0.5 mL), and the homogeneous solution was obtained after mixing and ultrasonication for 15 minutes. The above solution was cast uniformly onto an indium tin oxide coated plate and pristine film was obtained by evaporating the solvent in an oven (60℃). Fabrication of LZU1 membrane from pristine film: The pristine film on the indium tin oxide coated plate is placed in a mixed solution of tetrahydrofuran and aqueous acetic acid with a volume ratio of 6:4. After reacting at 65℃ for a certain period, LZU1 membrane was obtained.

***S1.6 Fabrication of LZU1 membrane via imine condensation reaction in the presence of aniline***

Fabrication of pristine film: TFB (0.04 mmol), PDA (0.06 mmol) and aniline (0.12 mmol) were dissolved in DMAC (1 mL), and the homogeneous solution was obtained after ultrasonication for 15 minutes. The above solution was cast uniformly onto an indium tin oxide coated plate and pristine film was obtained by evaporating the solvent in an oven (60℃). Fabrication of LZU1 membrane from pristine film: The pristine film on the indium tin oxide coated plate is placed in a mixed solution of tetrahydrofuran and aqueous acetic acid with a volume ratio of 6:4. After reacting at 65℃ for a certain period, LZU1 membrane was obtained.

***S1.7 Fabrication of LZU1 membrane via imine condensation reaction in the presence of aniline***

All calculations were performed using the DFT method implemented in the Gaussian 16 package. The molecular geometries of all structures were optimized by B3LYP functional and 6-31g (d) basis sets in the gas phase. In addition, all single-point energy and free energy calculations were performed using the B3LYP functional and 6-311G (d) basis sets. The sums of the electronic and thermal free energies of the reactants and products were calculated separately, and the reaction energy gap was determined on this basis.

**S2 Supporting Figures**


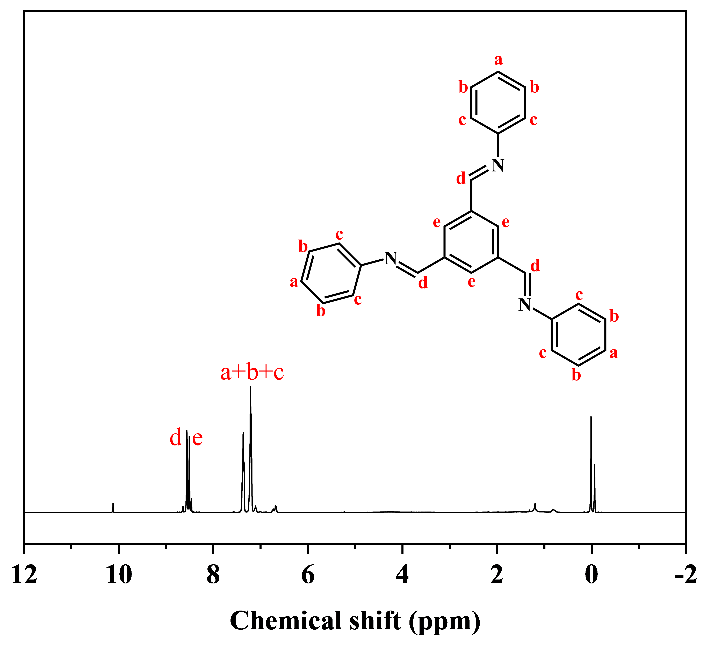


**Fig. S1** ^1^H NMR spectra (CDCl_3_, 400MHz) of BTMT.


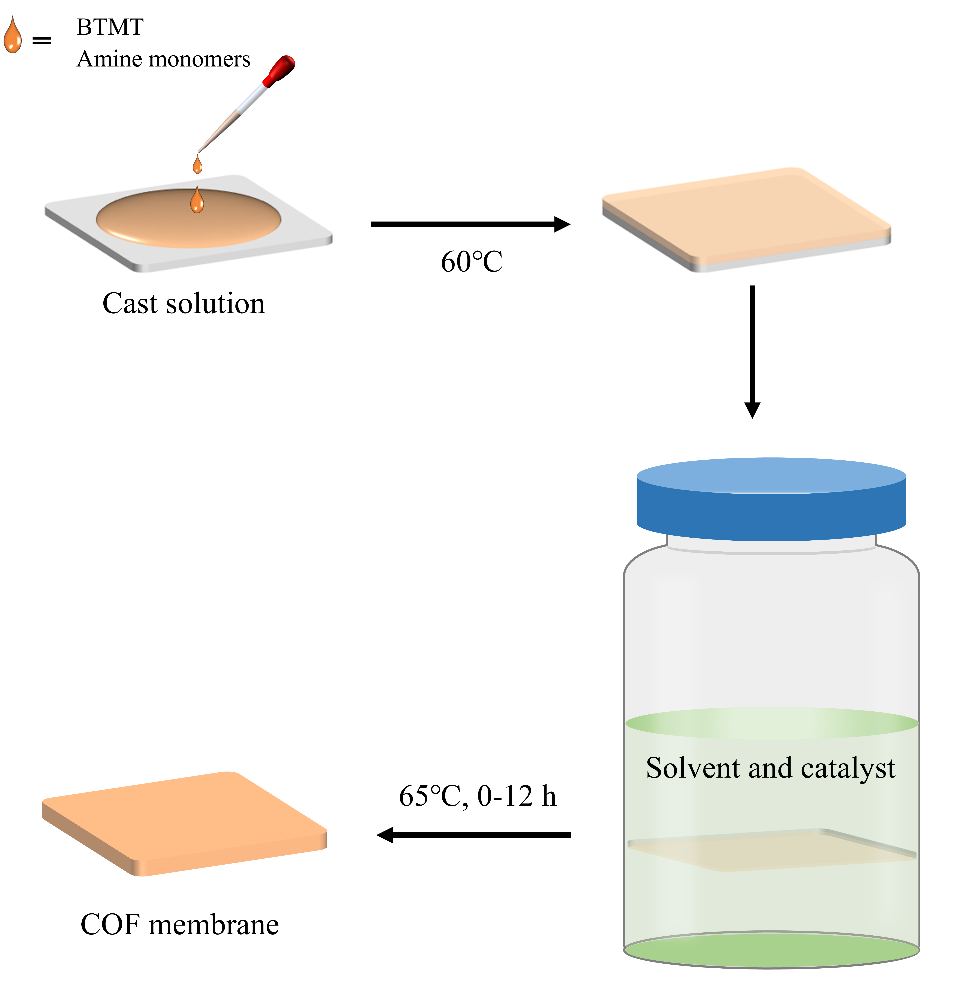


**Fig. S2** Fabrication process of IELZU1 membrane and IELZU1-PEI membrane.

**
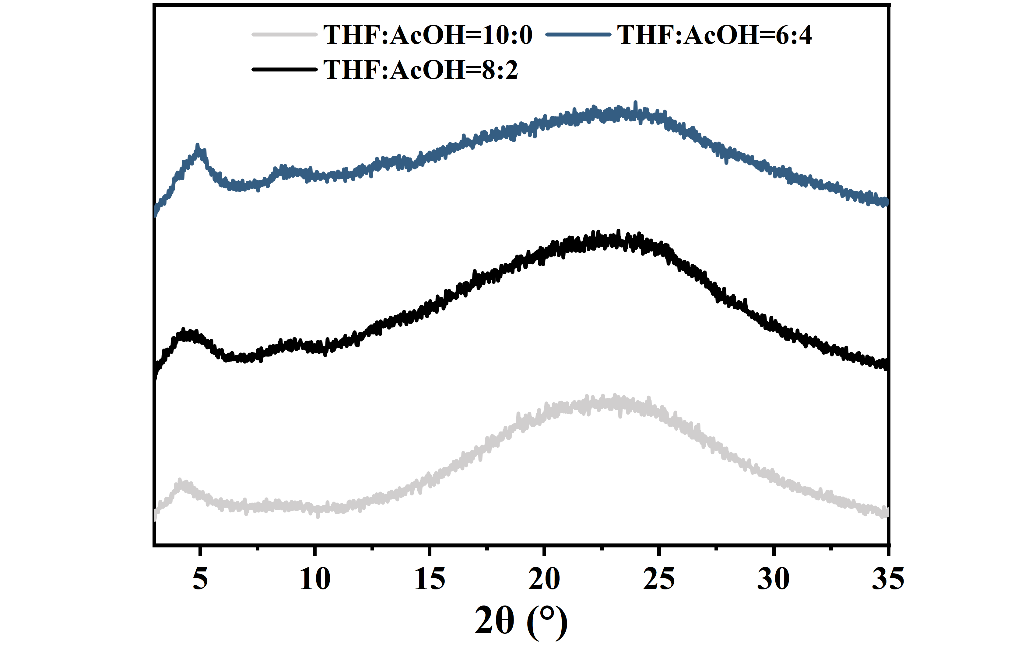
**

**Fig. S3** PXRD patterns of IELZU1 membrane: effect of solvent to catalyst ratios during imine exchange.

**
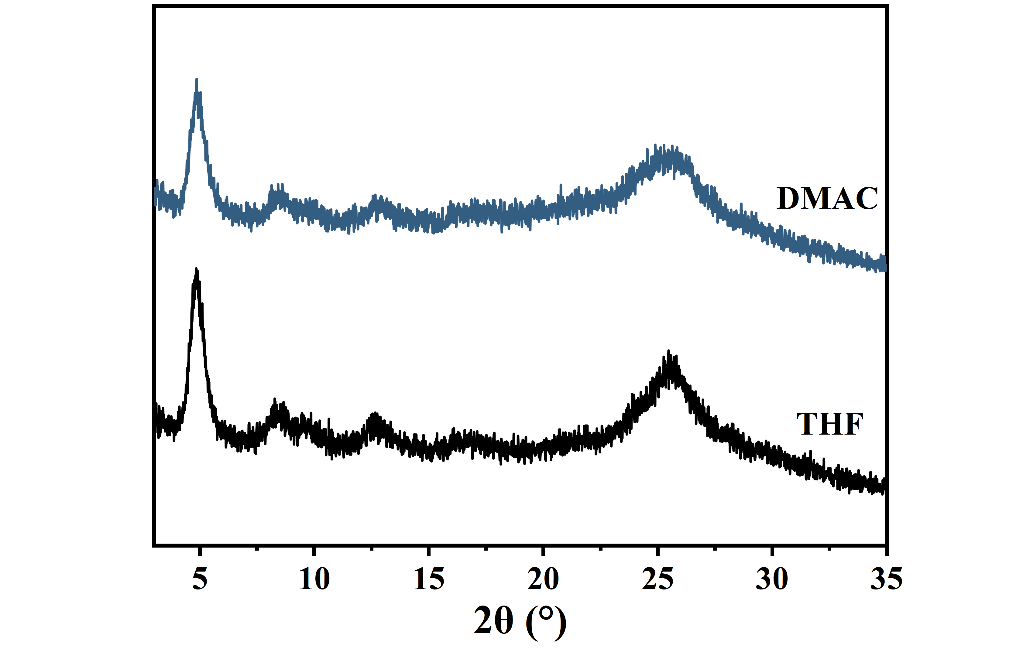
**

**Fig. S4** PXRD patterns of IELZU1 membrane: effect of different solvents during imine exchange reaction.


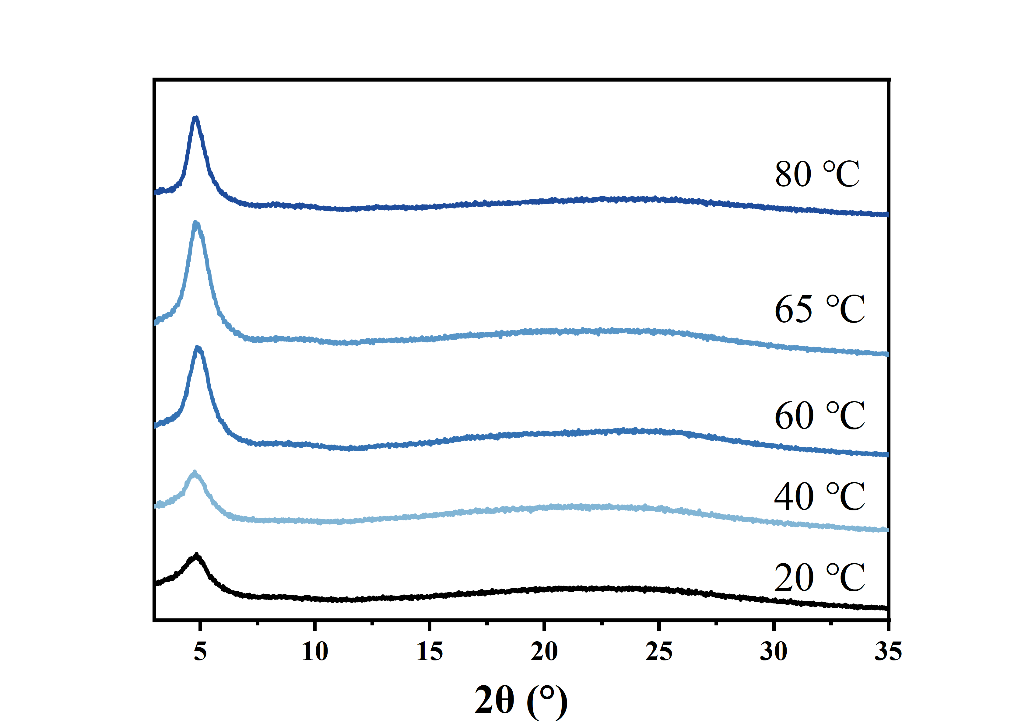


**Fig. S5** PXRD patterns of IELZU1 membrane: effect of different reaction temperature during imine exchange reaction.

**
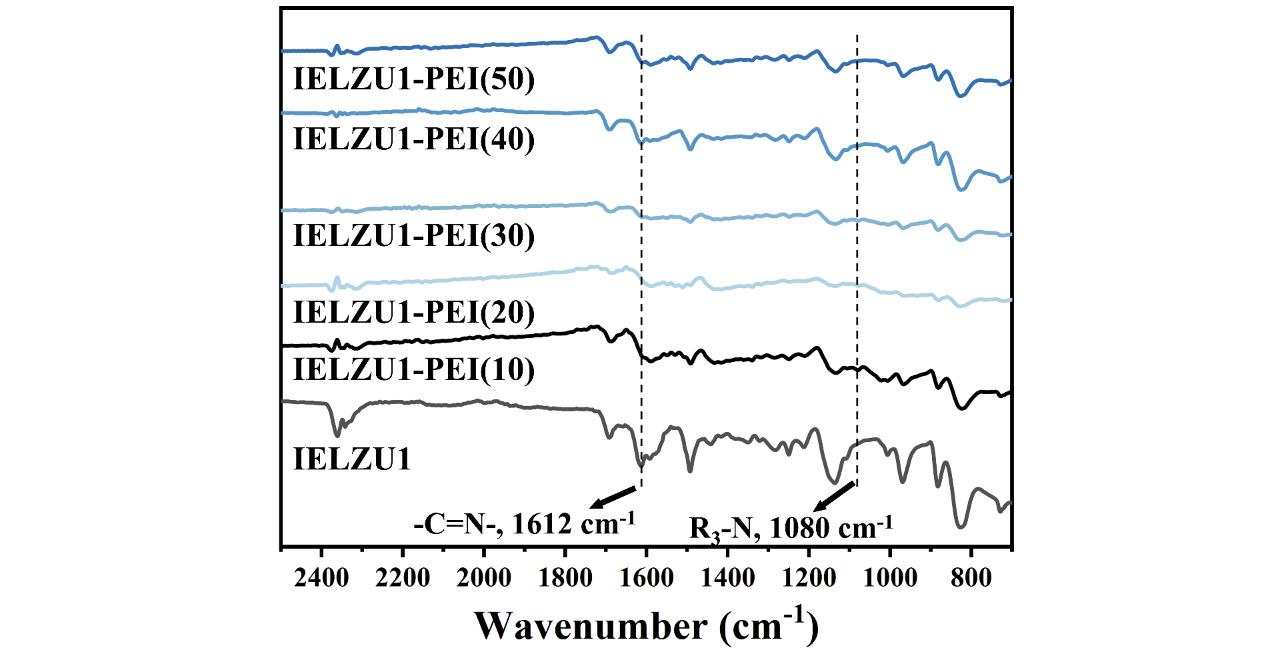
**

**Fig. S6** FTIR spectra of IELZU1 and IELZU1-PEI(x) membrane (x=10, 20, 30, 40 and 50 μL).


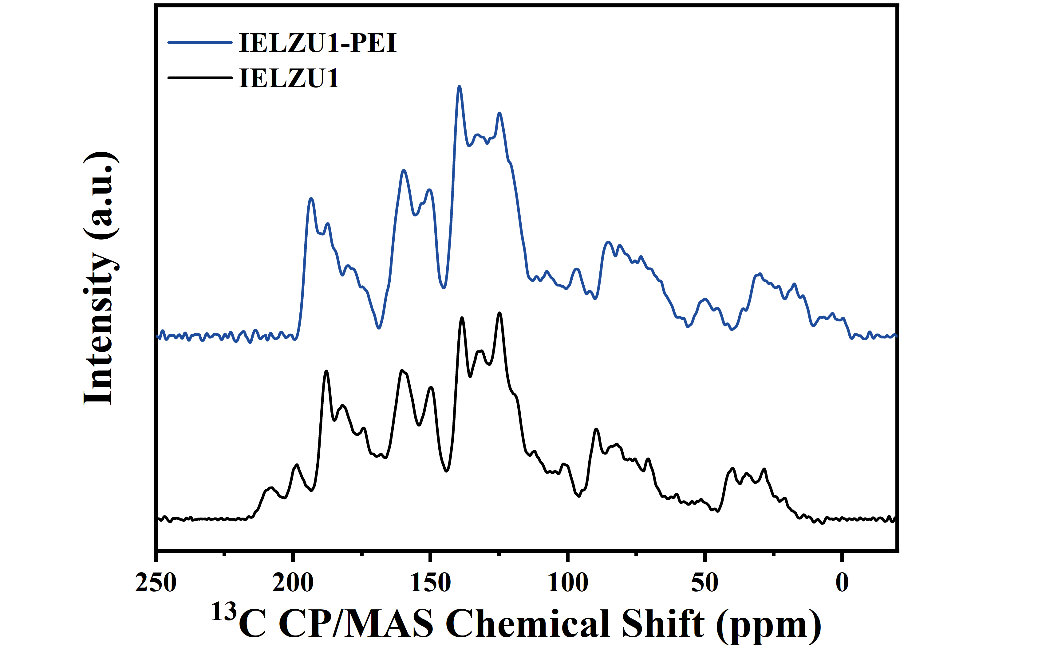


**Fig. S7** ^13^C CP/MAS sloid NMR spectra of IELZU1 membrane and IELZU1-PEI membrane. The IELZU1-PEI membrane presented in this figure was fabricated using 40 μL of PEI The emerged signals at 30 to 50 ppm in IELZU1-PEI spectrum indicated the successful incorporation of PEI in IELZU1-PEI membrane.

**
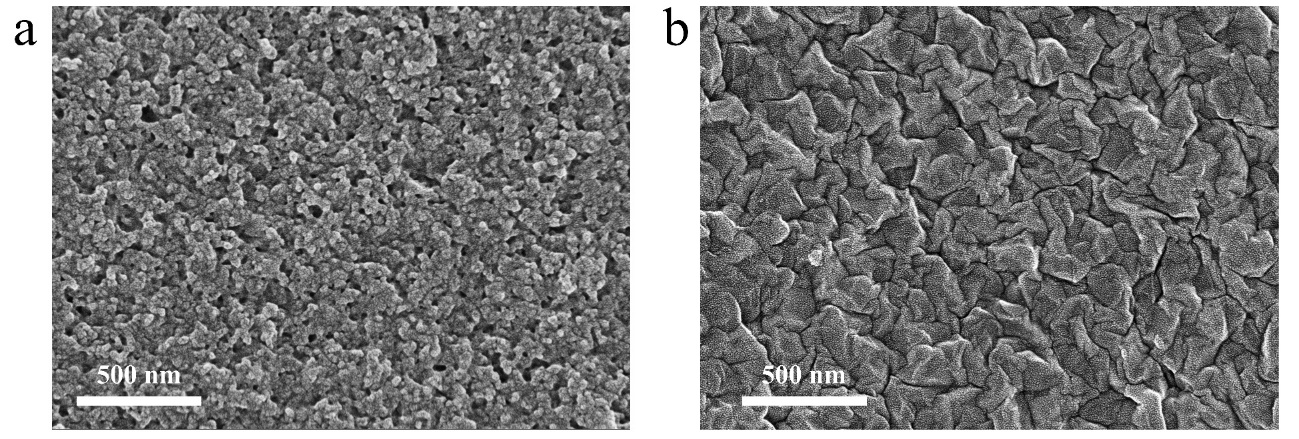
**

**Fig. S8** **a-b** SEM images of pristine film **(a)** and IELZU1 membrane **(b)**. Considering that the membrane had a membrane surface close to the indium tin oxide coated plate and a membrane surface evaporated by the solvent during the membrane fabrication process, their morphologies may be different, so we characterized both surfaces. The above figure shows the morphology of pristine film and IELZU1 membrane close to the surface of the solvent. The obvious change in the membrane morphology further indicated that the imine exchange reaction had occurred.

**
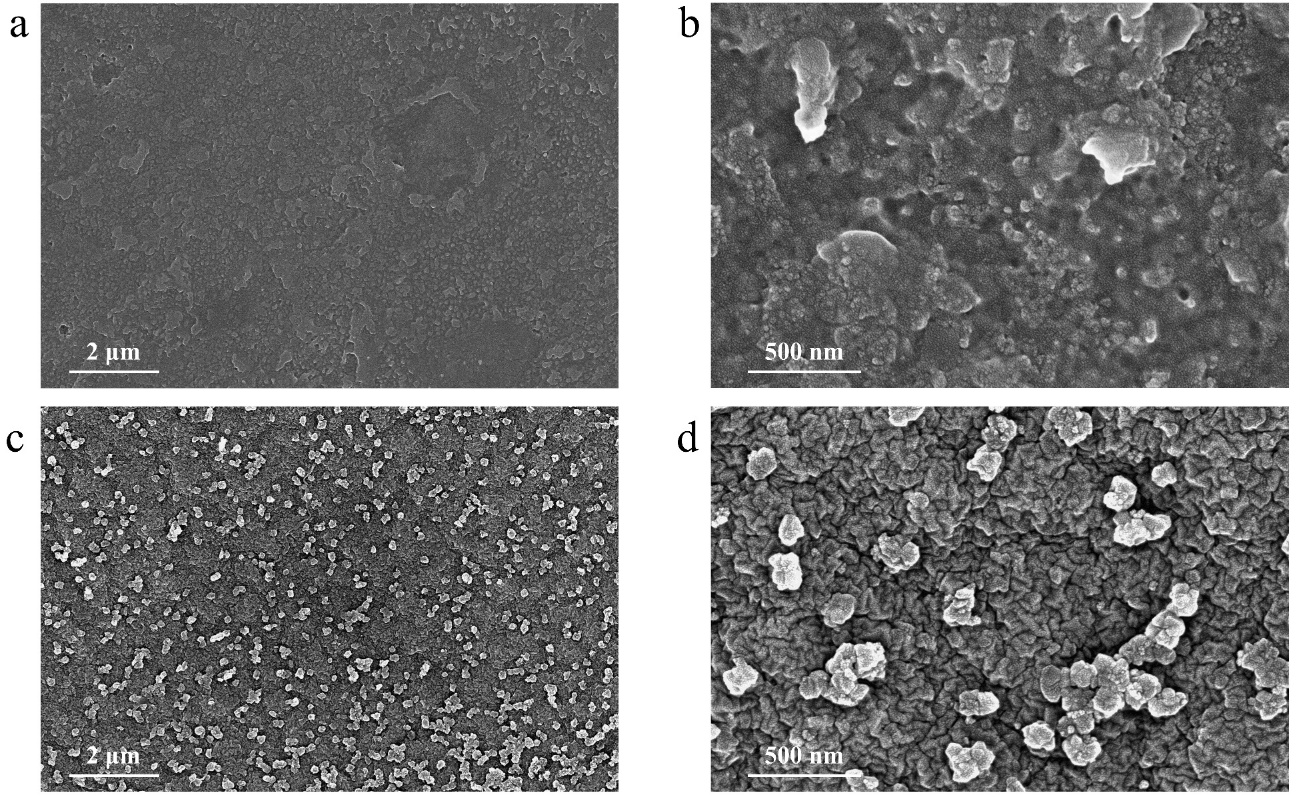
**

**Fig. S9** **a**-**d** SEM images of pristine film **(a-b)** and IELZU1 membrane **(c-d)**.The above figure shows the morphology of the pristine film and the COF membrane close to the surface of the indium tin oxide coated plate.


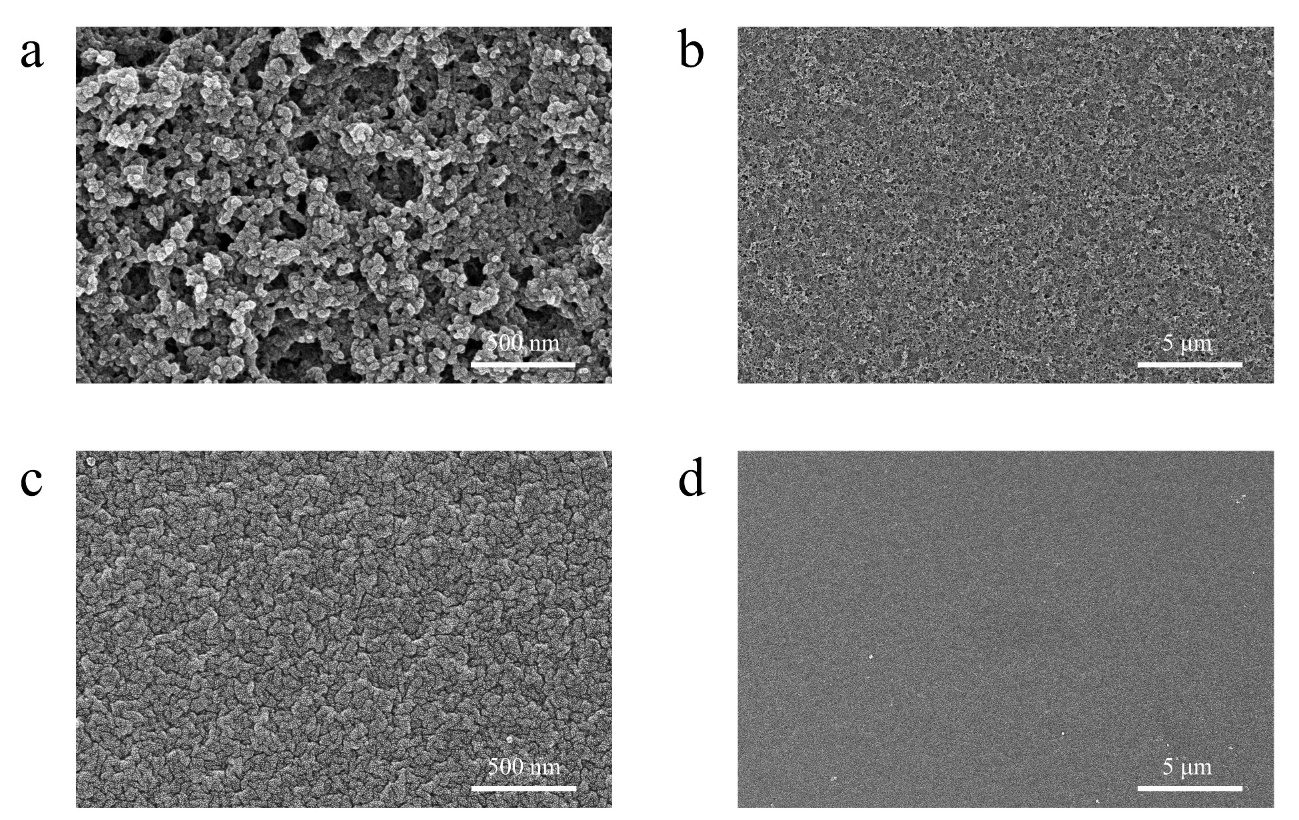


**Fig. S10** **a**-**d** SEM images of IELZU1-PEI membrane close to the surface of the solvent **(a-b)** and close to the indium tin oxide coated plate **(c-d)**. The amount of PEI used in IELZU1-PEI membrane shown in this figure is 40 μL.


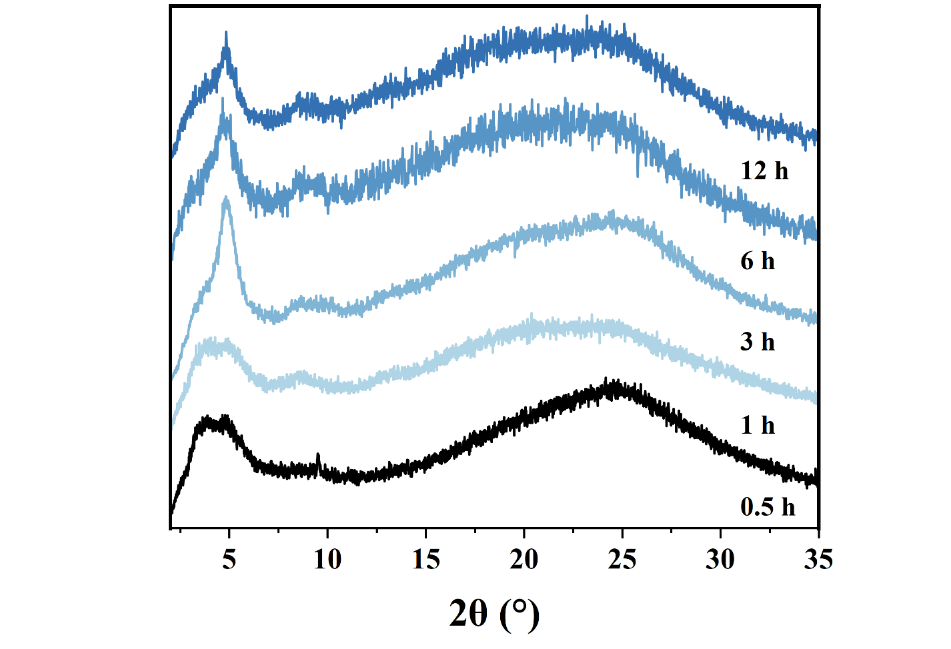


**Fig. S11** PXRD patterns of IELZU1-PEI membrane at different imine exchange times. The IELZU1-PEI membrane presented in this figure was fabricated using 40 μL of PEI.


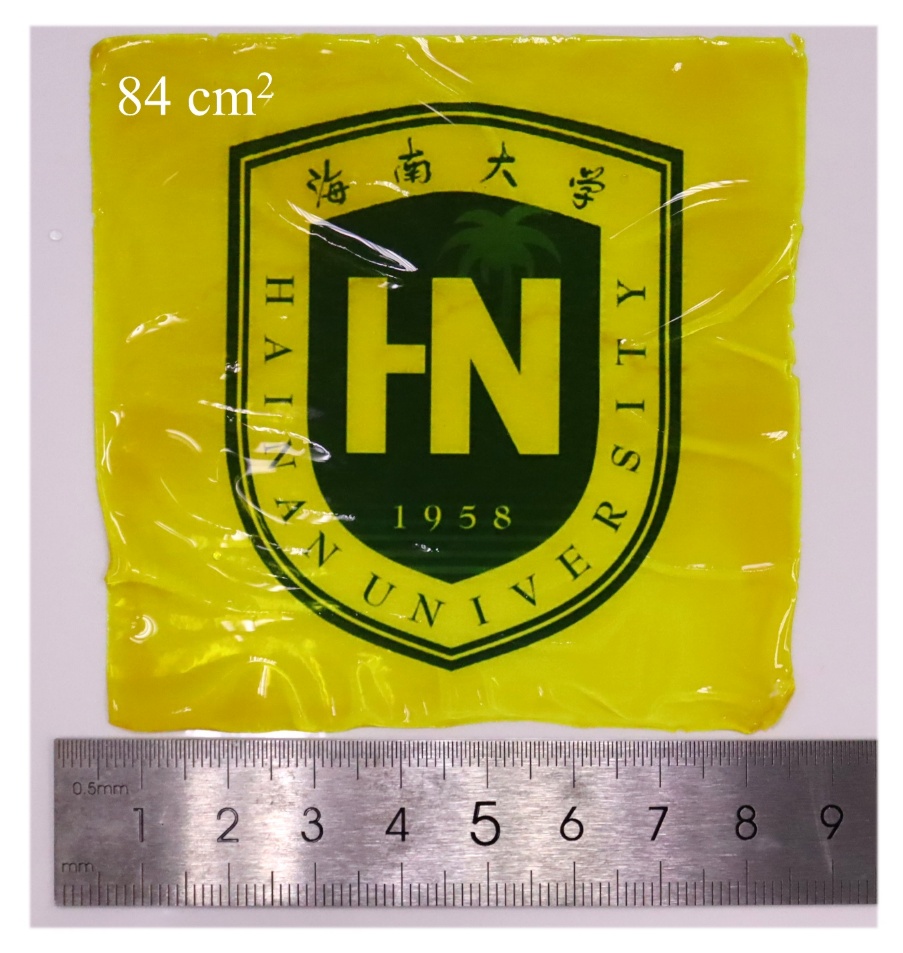


**Fig. S12** Optical image of IELZU1-PEI membrane fabricated in a large area (the membrane area is approximately 84 cm^2^). The IELZU1-PEI membrane presented in this figure was fabricated using 40 μL of PEI.

**
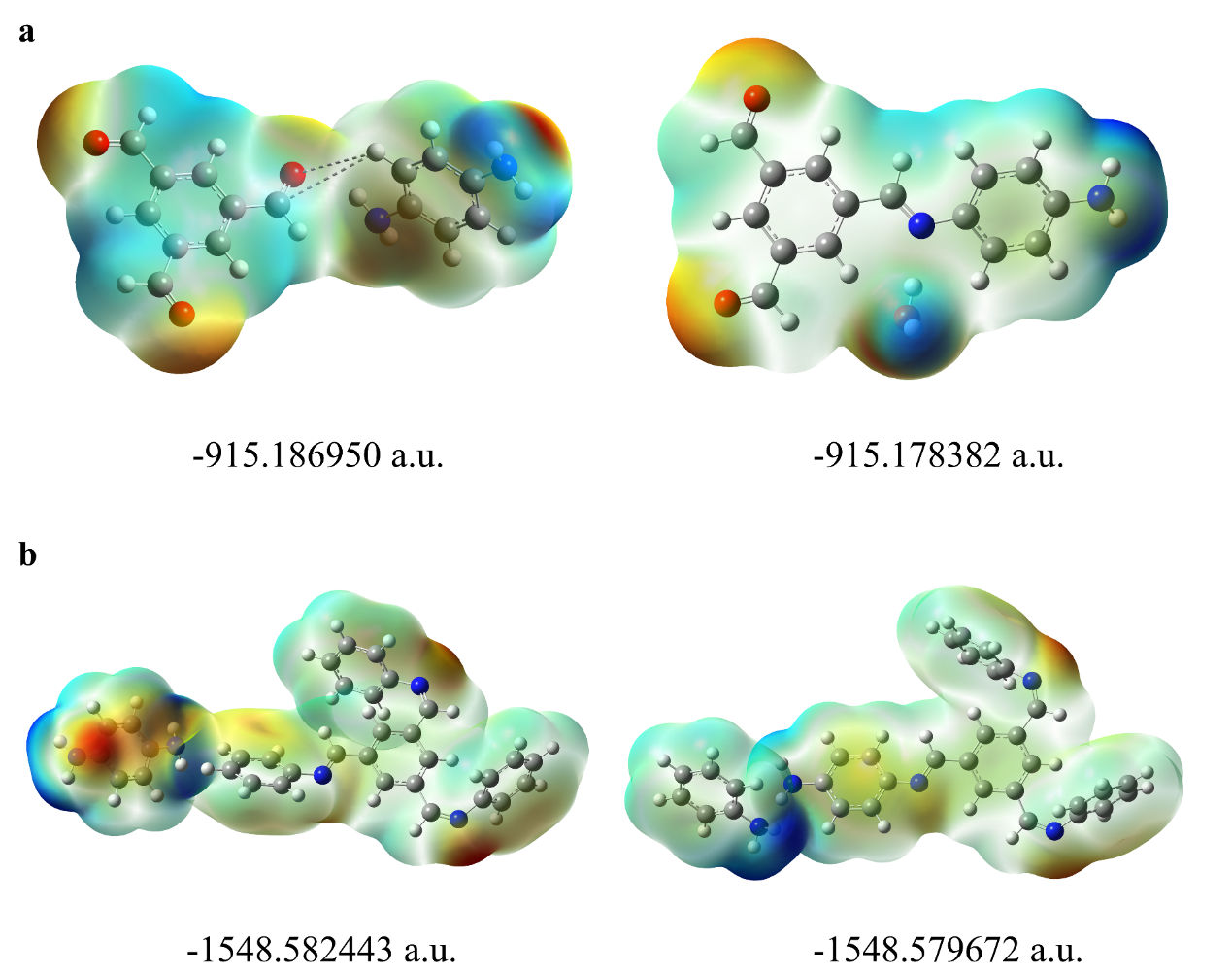
**

**Fig. S13** The total energy gap between the reactants and products of imine condensation reaction and imine exchange reaction (for aromatic amine) was obtained through DFT calculations [S3]. Energies below conformers were the sum of electronic and thermal free energies (gas phase conformers). The corresponding energy gap between reactants and products was shown in the figures. For the imine condensation reaction, the energy gap between the reactants and the products was 5.46 kcal/mol, whereas for the imine exchange reaction, the energy gap between the reactants and the products was 1.77 kcal/mol.

**
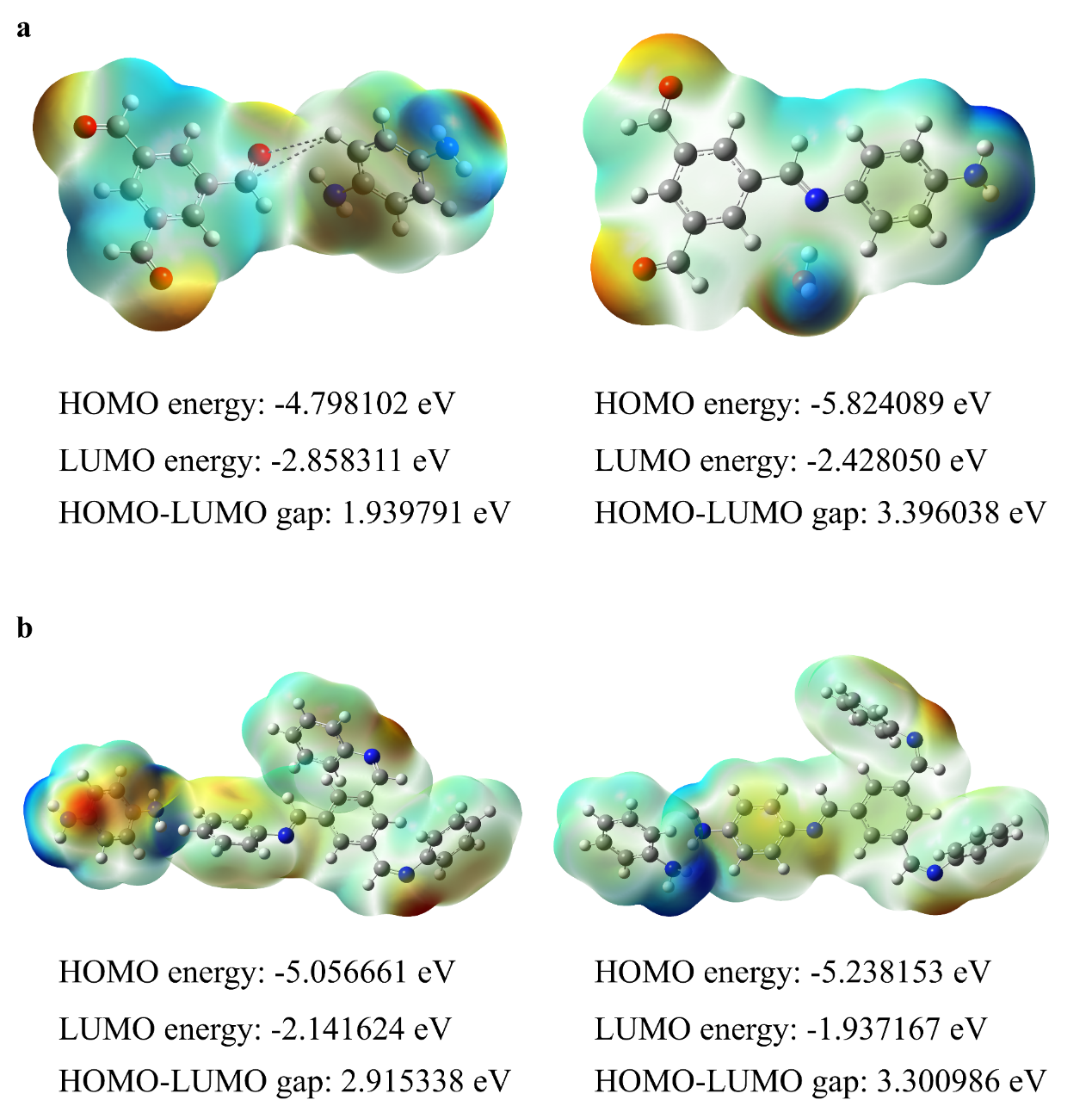
**

**Fig. S14** The total energy gap between the reactants and products of imine condensation reaction and imine exchange reaction (for aromatic amine) was obtained through DFT calculations.


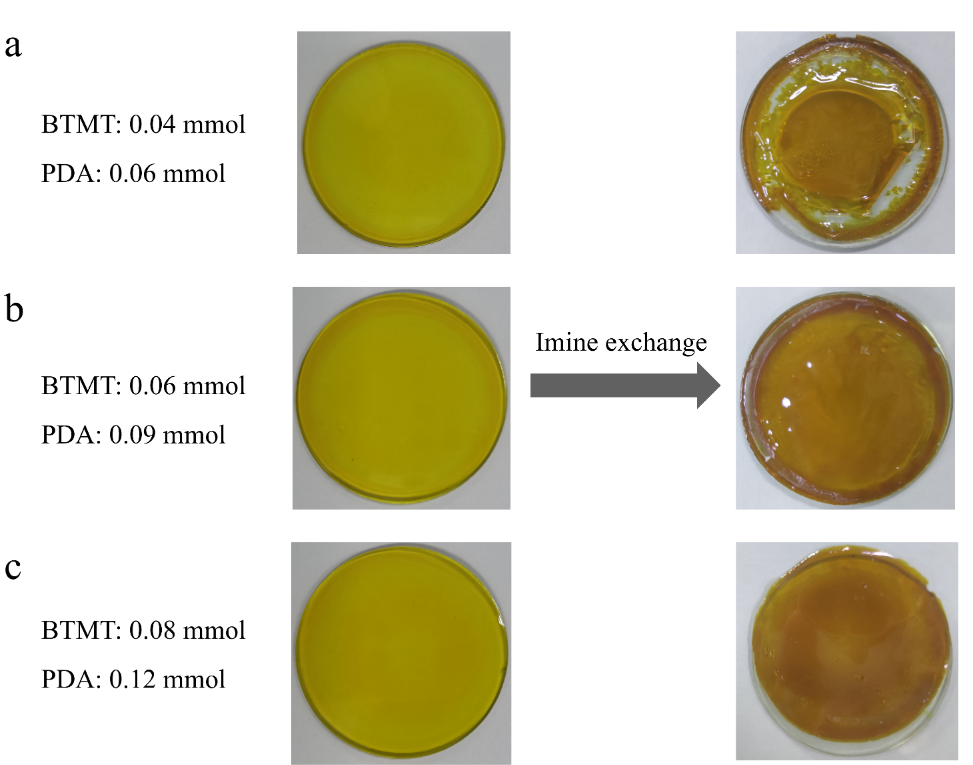


**Fig. S15** Optical images of pristine film and COF membranes at different BTMT and PDA concentrations.


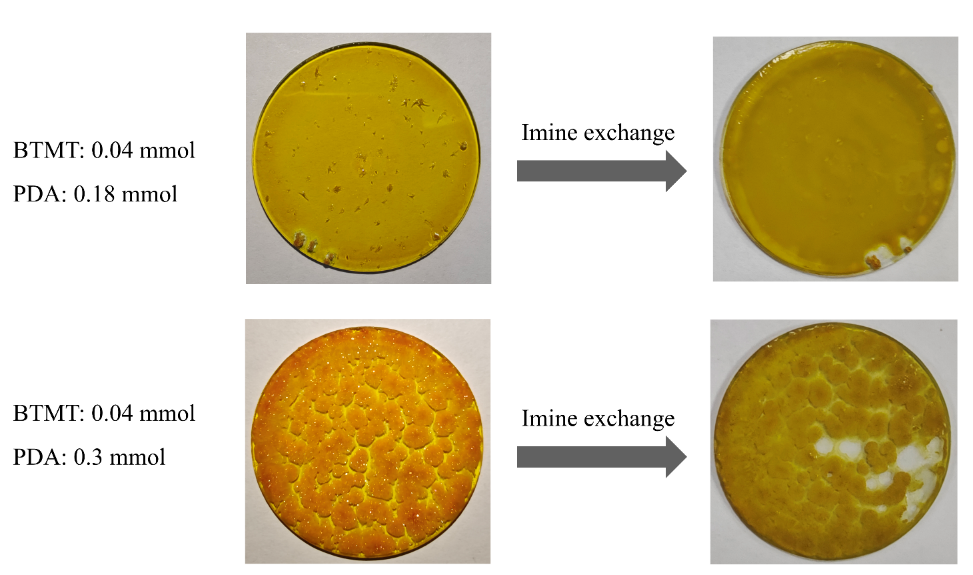


**Fig. S16** Optical images of the pristine films and the corresponding COF membranes were prepared with different PDA concentrations while keeping the BTMT concentration constant.


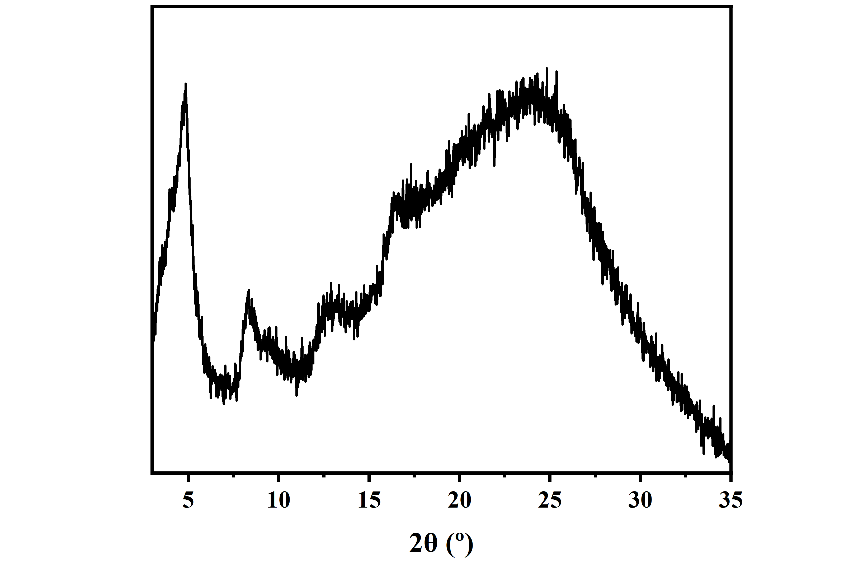


**Fig. S17** PXRD pattern of IELZU1 membrane at increasing PDA ratios (BTMT: 0.04 mmol, PDA: 0.12 mmol). Increasing the PDA concentration in the casting solution was conducive to enhancing the crystallinity of the COF membranes (considering that when the amount of PDA was increased to 0.3 mmol, discontinuous powder accumulation was obtained after solvent evaporation, and its XRD data were not tested).


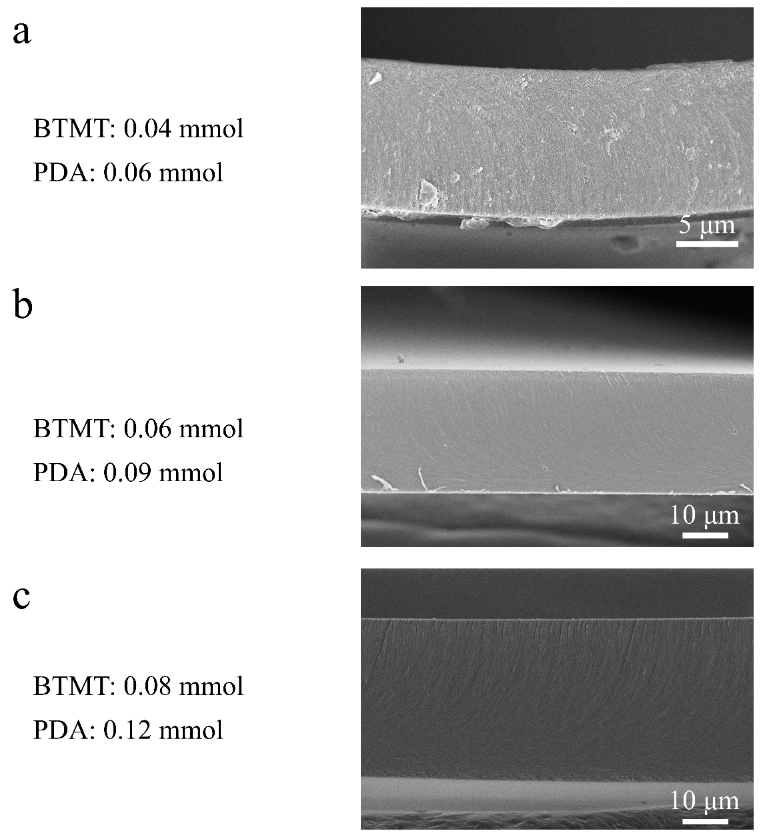


**Fig. S18** Cross-sectional SEM image of IELZU1 membranes at different BTMT and PDA concentrations.


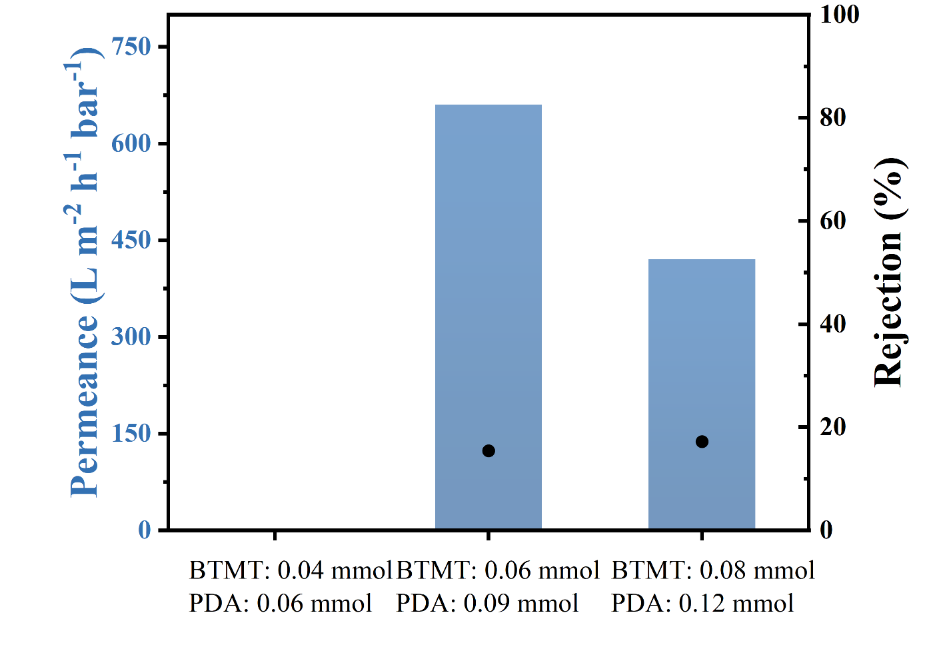


**Fig. S19** Permeance and CR rejection of IELZU1 membranes fabricated at different concentrations. The IELZU1 membrane, prepared with a lower casting concentration (BTMT: 0.04 mmol, PDA:0.06 mmol), did not support nanofiltration testing. As shown in Supplementary Figs. 17 and 18, IELZU1 membrane exhibited a water permeance of 66 L·m^-2^·h^-1^·bar^-1^, while the rejection for Congo red, used as a model dye, was 15%. The low rejection implied that there were more defect sites within the membrane, which may originate from the failure to fully fill the sites occupied by aniline after it flowed out of the film from the amorphous structure to the framework structure. Simply increasing the membrane thickness did not achieve defect remedy; instead, it reduced the water permeance due to the increased thickness. This indicated that the membranes fabricated through the imine exchange reaction had intrinsic defects, which were independent of their thickness.

**
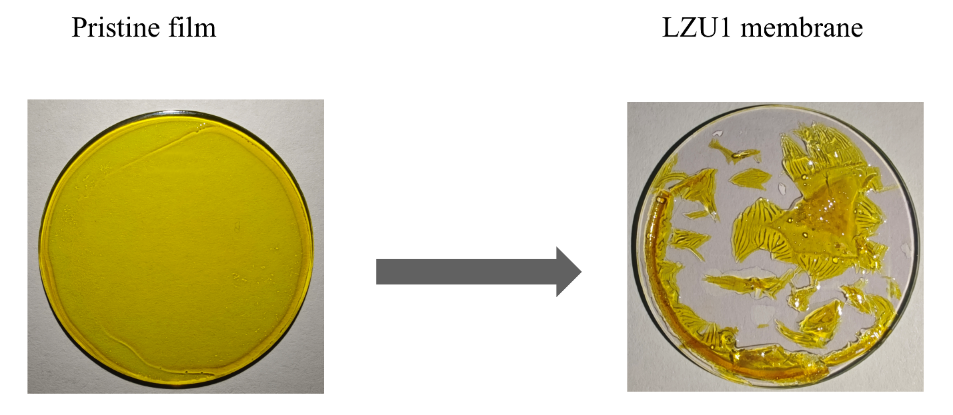
**

**Fig. S20** Optical images show pristine film and the resulting LZU1 membrane fabricated via imine condensation reaction.

**
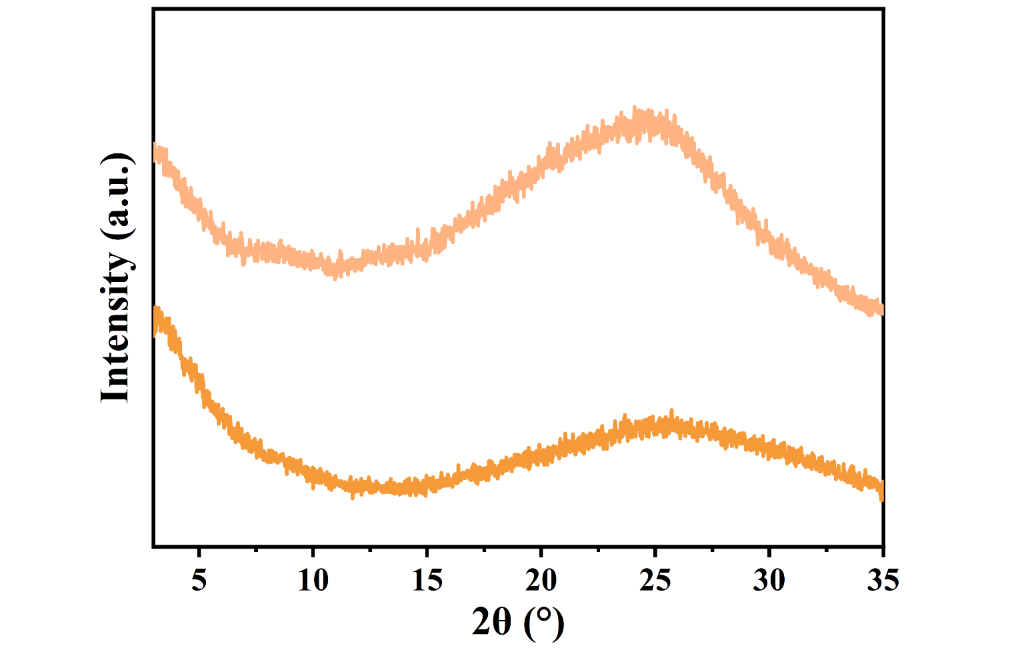
**

**Fig. S21** PXRD patterns of LZU1 membranes fabricated via imine condensation reaction. The upper curve represents the LZU1 membrane and the lower curve represents the pristine film.

**
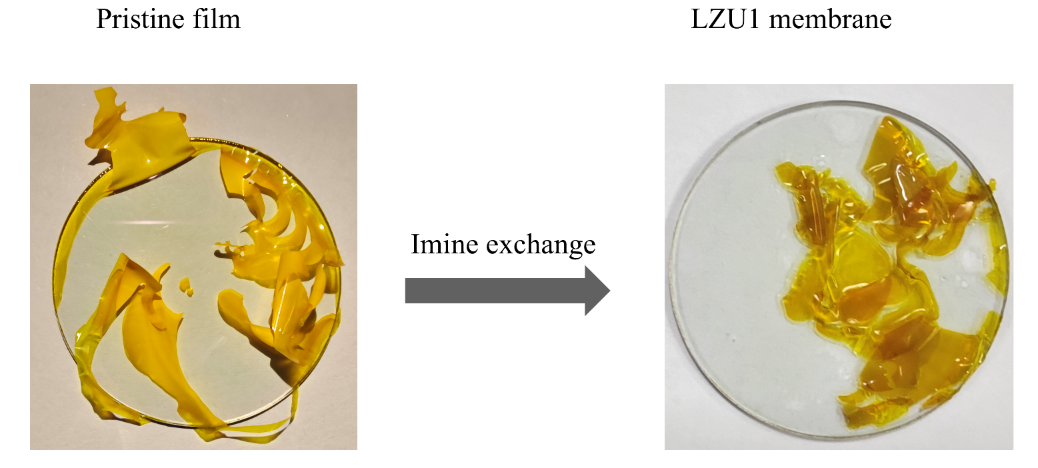
**

**Fig. S22** Optical images show pristine film and the resulting LZU1 membrane fabricated via imine condensation reaction in the presence of aniline.


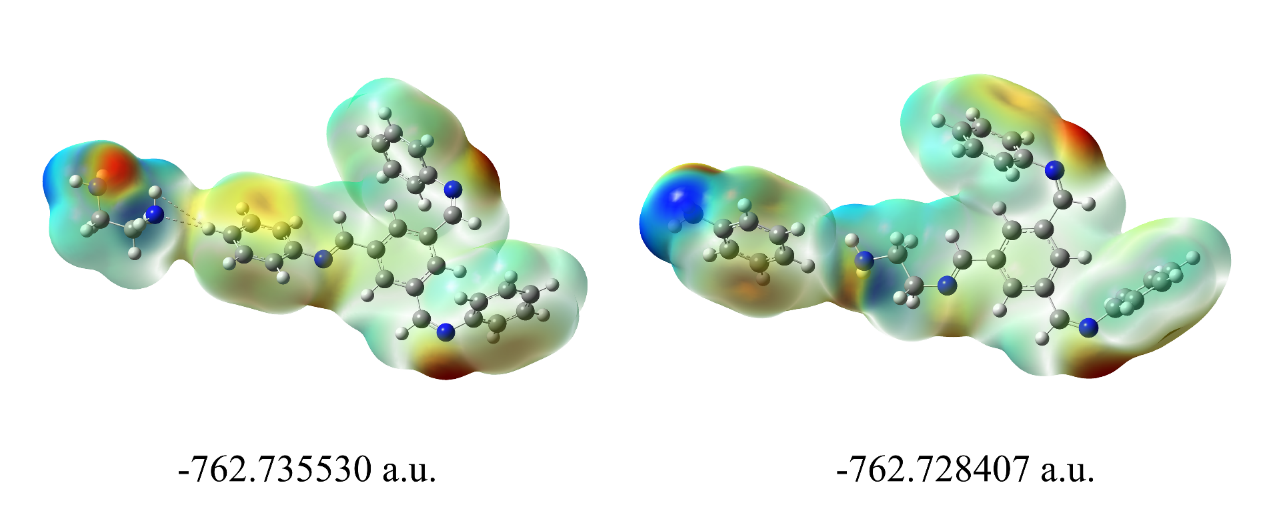


**Fig. S23** The total energy gap between the reactants and products of imine exchange reaction (for aliphatic amine) were obtained through DFT calculations (to reduce computational cost, ethylenediamine is used as a representative of PEI to undergo imine exchange with BTMT) [S4]. Energies below conformers were the sum of electronic and thermal free gap (gas phase conformers). The corresponding energy gaps between reactants and products are shown in the figure. The total energy gap between the reactants and the products was 4.54 kcal/mol.


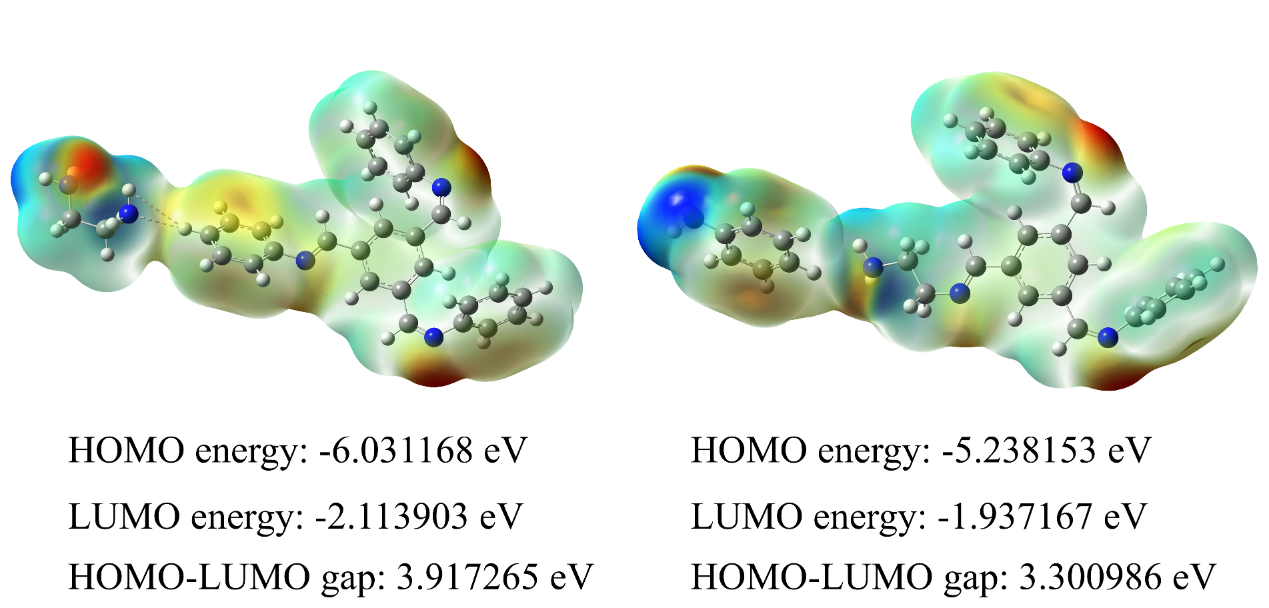


**Fig. S24** The energy gap between the reactants and products of imine exchange reaction (for aliphatic amine) was obtained through DFT calculations.


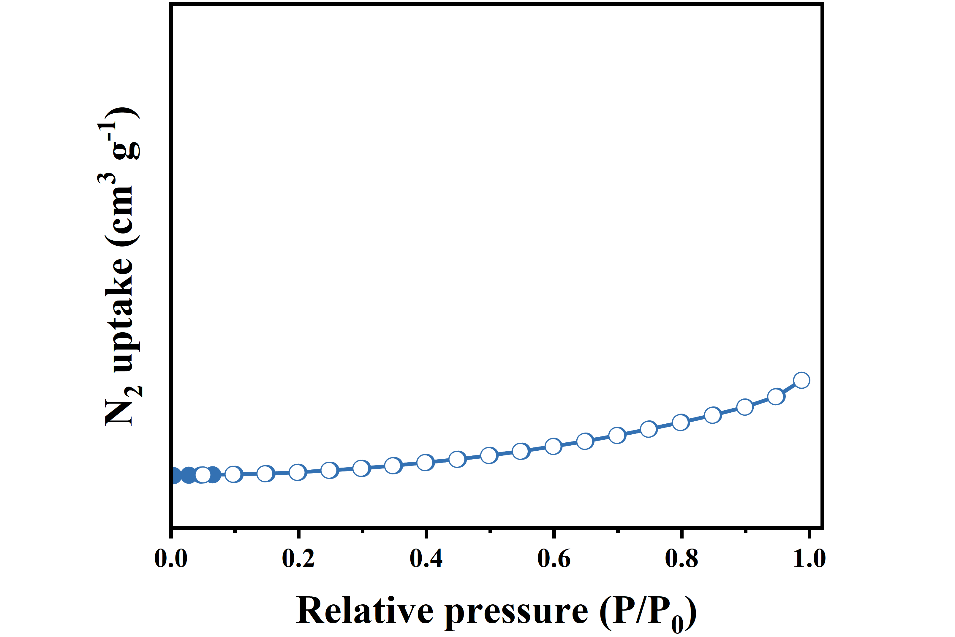


**Fig. S25** N_2_ adsorption/desorption isotherms at 77 K for IELZU-PEI(50) membrane.


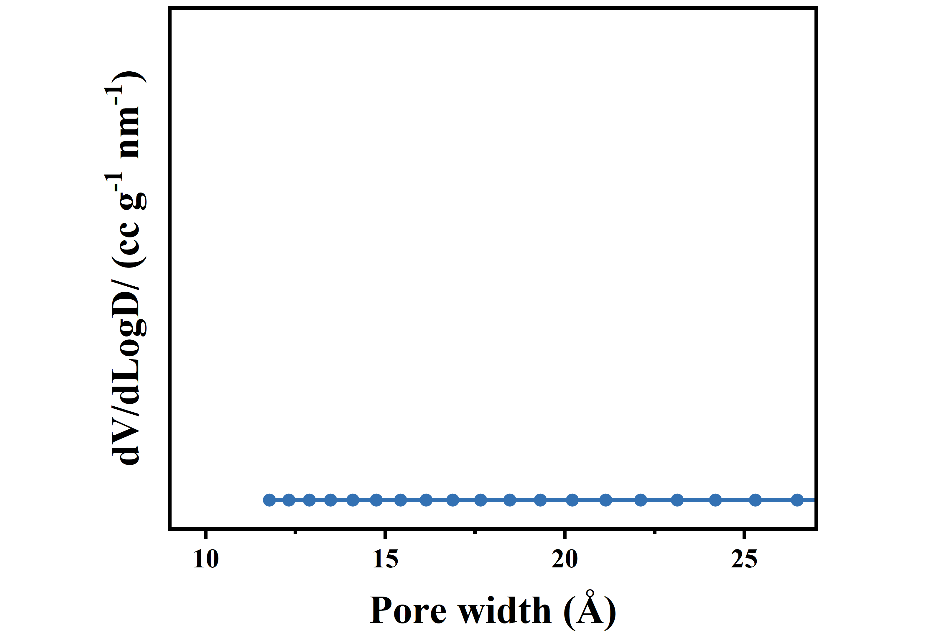


**Fig. S26** Pore size distribution of IELZU-PEI(50) membrane. (The pore size distribution calculated through the nonlocal density functional theory (NLDFT) method).


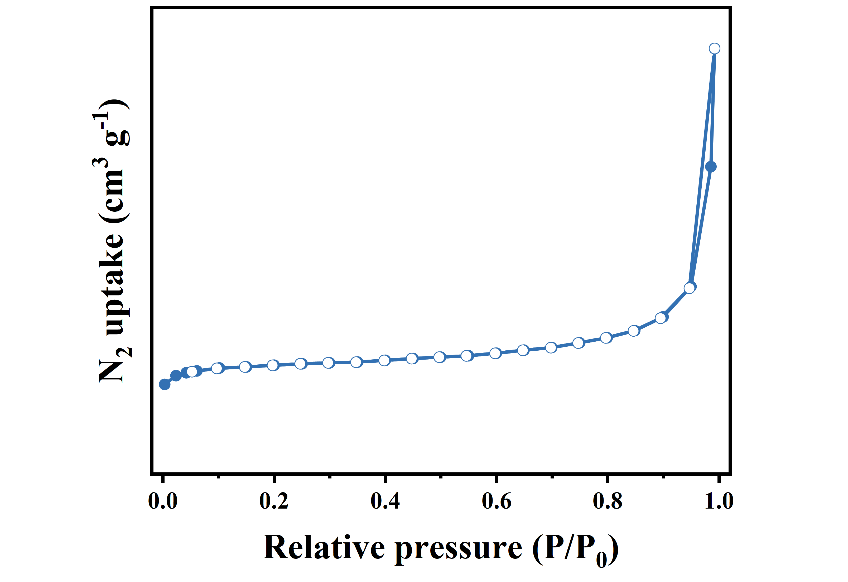


**Fig. S27** N_2_ adsorption/desorption isotherms at 77 K for IELZU1 powder.


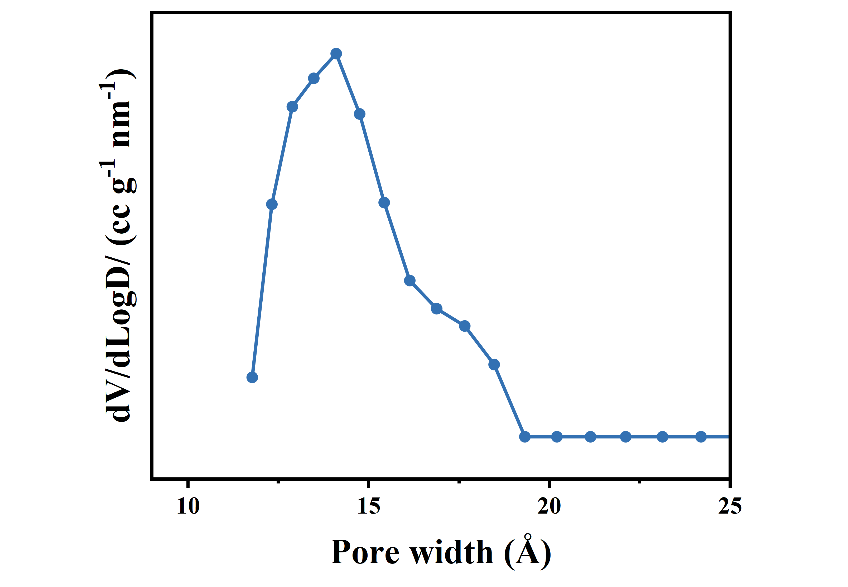


**Fig. S28** Pore size distribution of IELZU1 powder (the unimodal pore size distribution calculated through the non-logical density functional theory (NLDFT) method). The pore size distribution of IELZU1 powder was similar to that of the IELZU1 membrane and is consistent with previous literature reports.


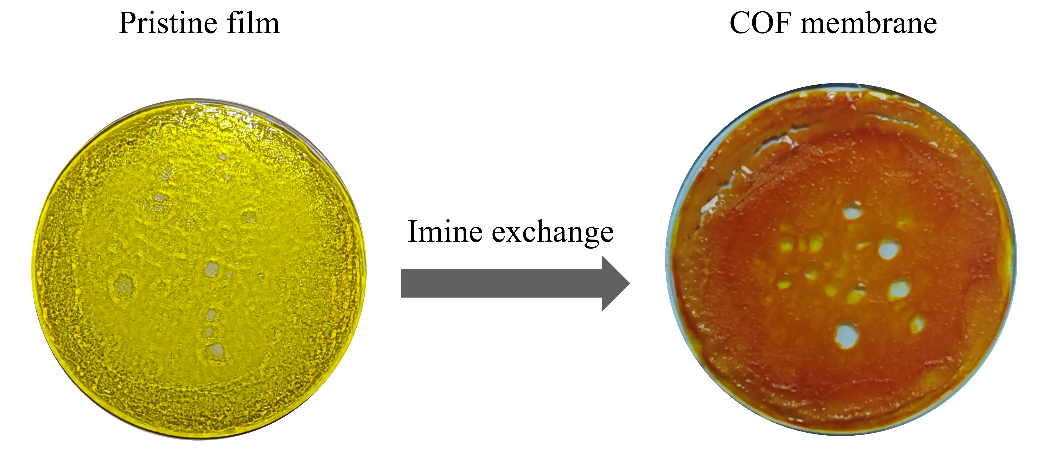


**Fig. S29** Optical images show pristine film and the resulting COF membrane fabricated via imine exchange reaction with linear PEI.


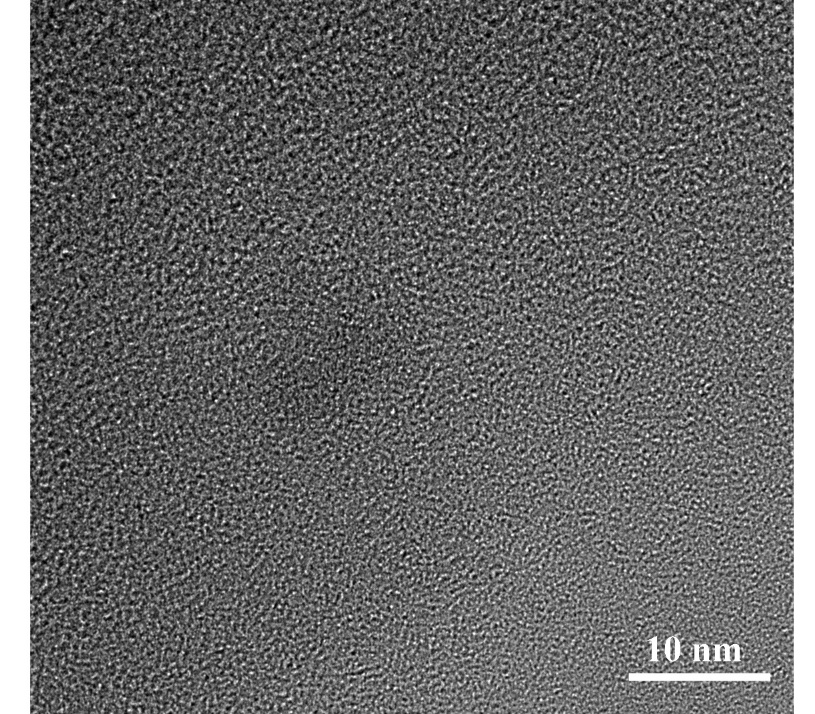


**Fig. S30** TEM images of IELZU1-PEI(50) membrane.


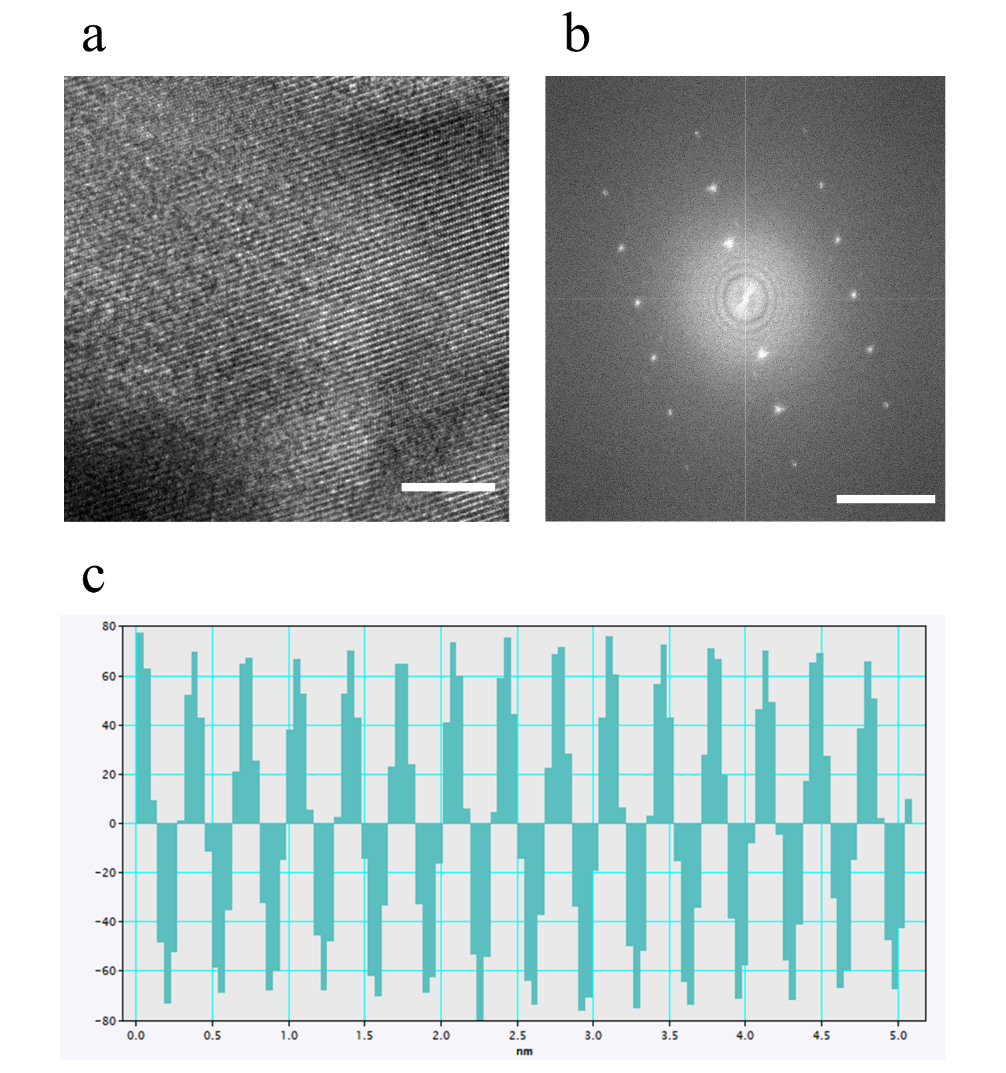


**Fig. S31** **a-b** Enlarged TEM images of IELZU1 membrane **a** and corresponding FFT pattern **b**. **c** The interplanar spacing of the (001) crystal plane of the IELZU1 membrane was measured from the TEM image to be 0.339 nm. Scale bar: 5 nm **a** and 5 /1 nm **b**. The TEM of IELZU1 membrane shows an interlayer spacing of approximately 0.339 nm for the (001) facet, a value similar to those reported in the literature [S4]. However, clear boundaries between highly crystalline and low-crystalline regions can be distinctly observed from the image. This indicates that IELZU1 membrane has a significant number of intermolecular defects.


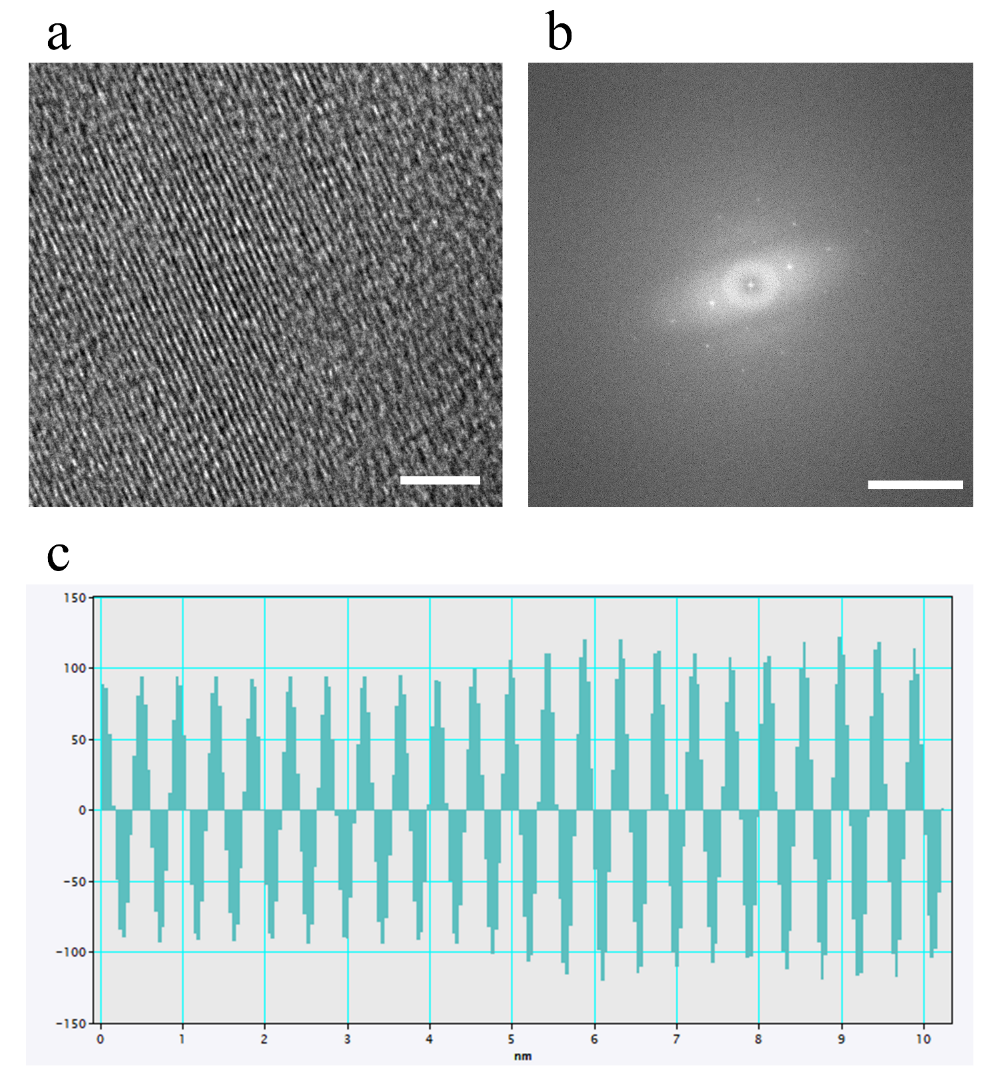


**Fig. S32** **a-b** Enlarged TEM images of IELZU1-PEI(10) membrane **a** and corresponding FFT pattern **b**. **c** The interplanar spacing of the (001) crystal plane of the IELZU1 membrane was measured from the TEM image to be 0.447 nm. Scale bar: 5 nm **a** and 5 /1 nm **b**.


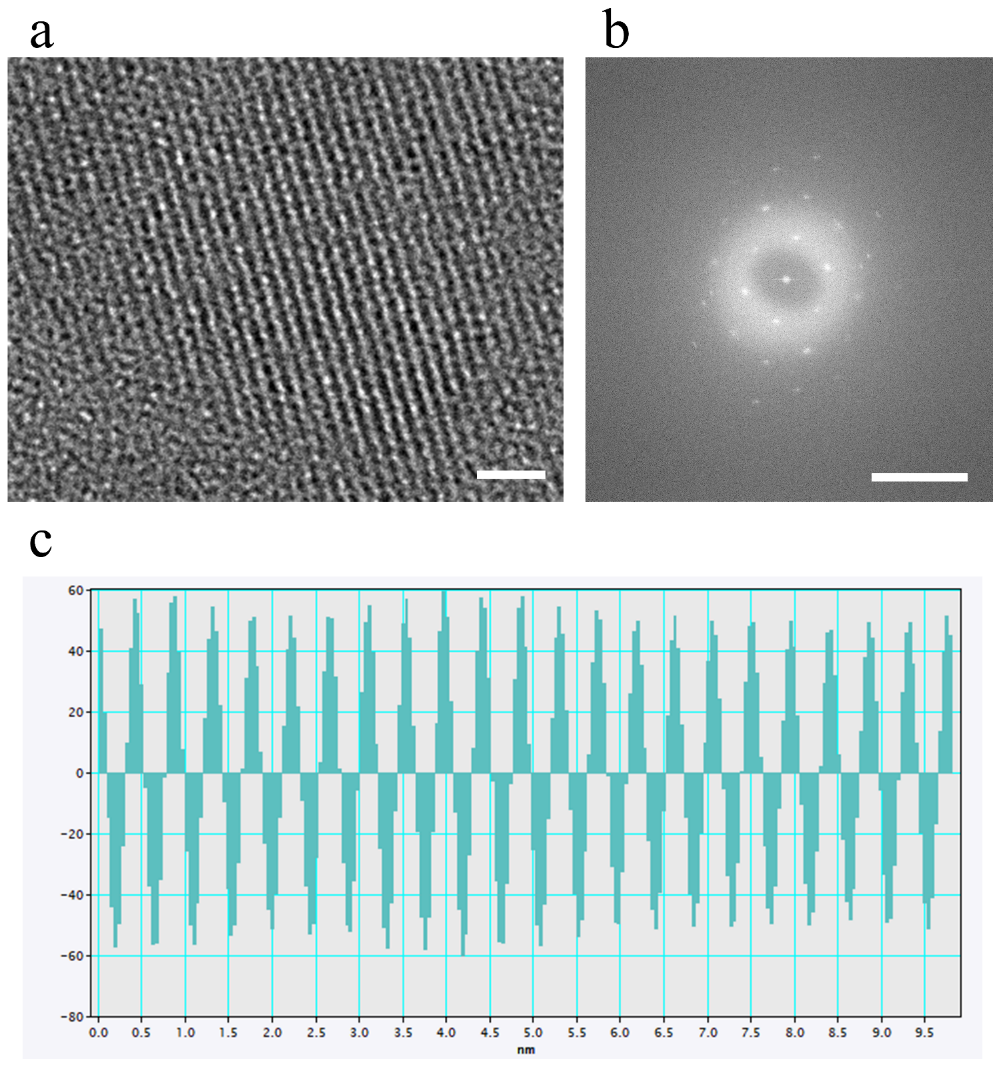


**Fig. S33** **a-b** Enlarged TEM images of IELZU1-PEI(20) membrane **a** and corresponding FFT pattern **b**. **c** The interplanar spacing of the (001) crystal plane of the IELZU1 membrane was measured from the TEM image to be 0.441 nm. Scale bar: 5 nm **a** and 5 /1 nm **b**.


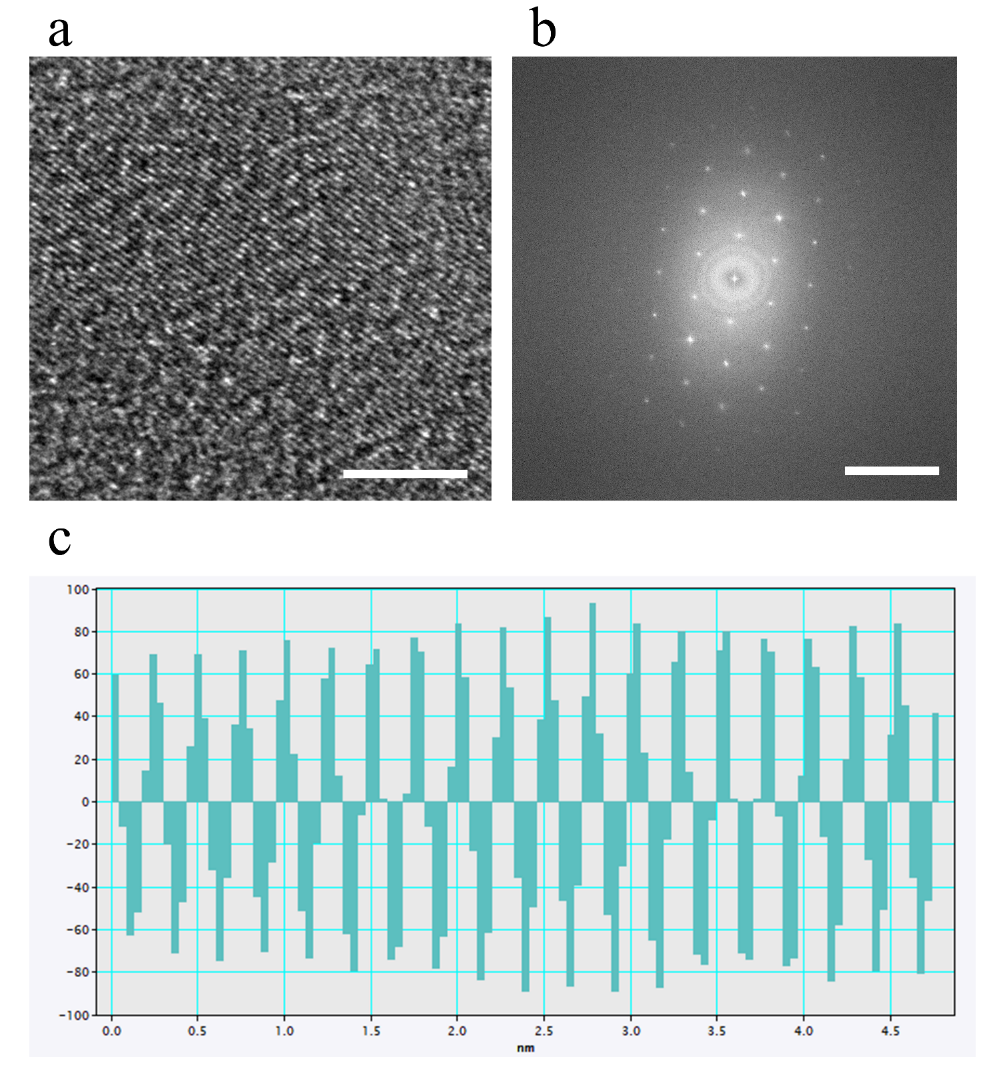


**Fig. S34** **a-b** Enlarged TEM images of IELZU1-PEI(40) membrane **a** and corresponding FFT pattern **b**. **c** The interplanar spacing of the (001) crystal plane of the IELZU1 membrane was measured from the TEM image to be 0.252 nm. Scale bar: 5 nm **a** and 5 /1 nm **b**.


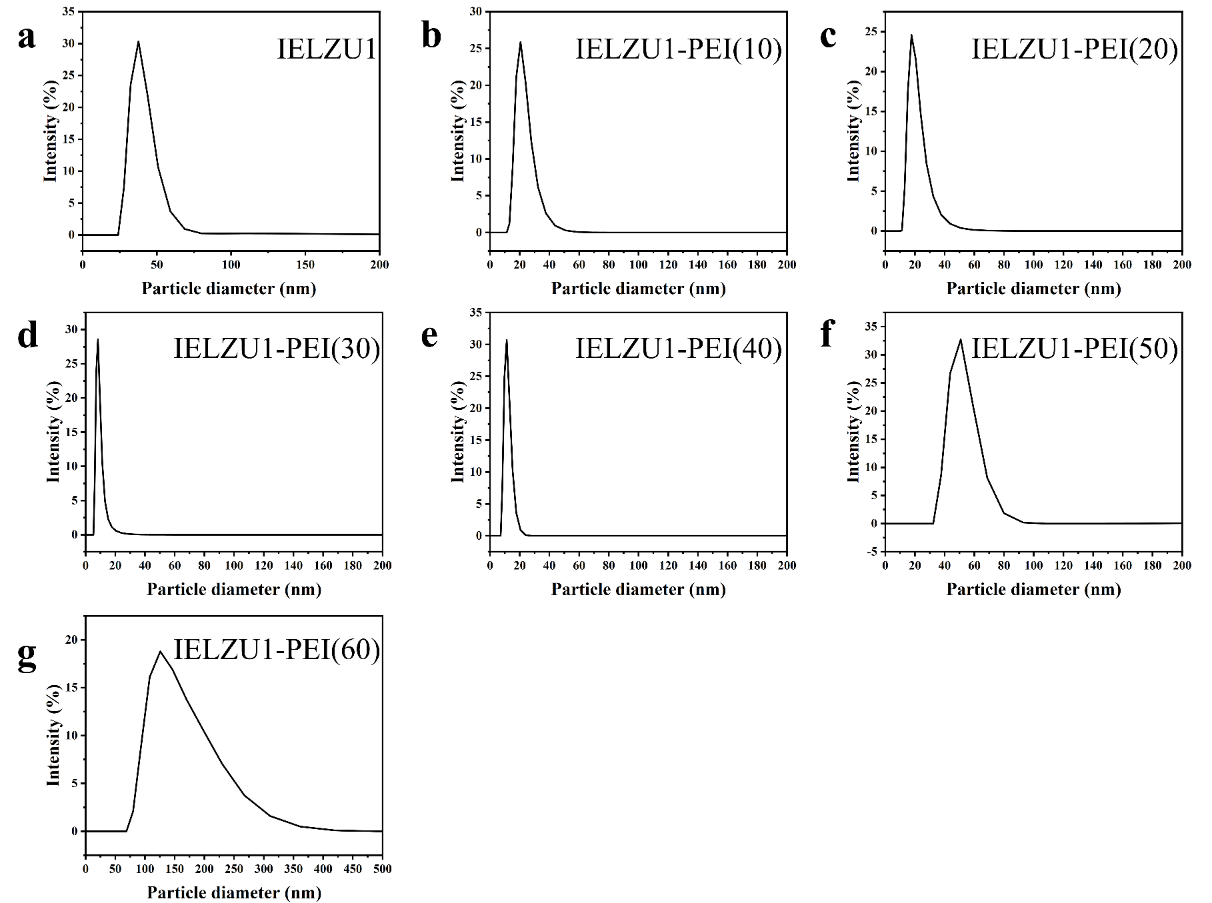


**Fig. S35** DLS data of casting solution at different PEI amounts. The amount of PEI added was a critical factor governing the membrane-formation behavior and crystallinity of COF membranes. Firstly, a low addition (0-20 μL) provided weak regulation, resulting in inhomogeneous crystal distribution and decreased membrane crystallinity. Secondly, an excessive amount (50-60 μL) exhibited over-regulation; it lowered the fraction of COF particles and induced over-entanglement, forming large particles with poor size distribution. Finally, an optimal addition (30-40 μL) enabled moderate regulation. This approach successfully enhanced crystal dispersion to improve film quality while simultaneously ensuring satisfactory membrane crystallinity.


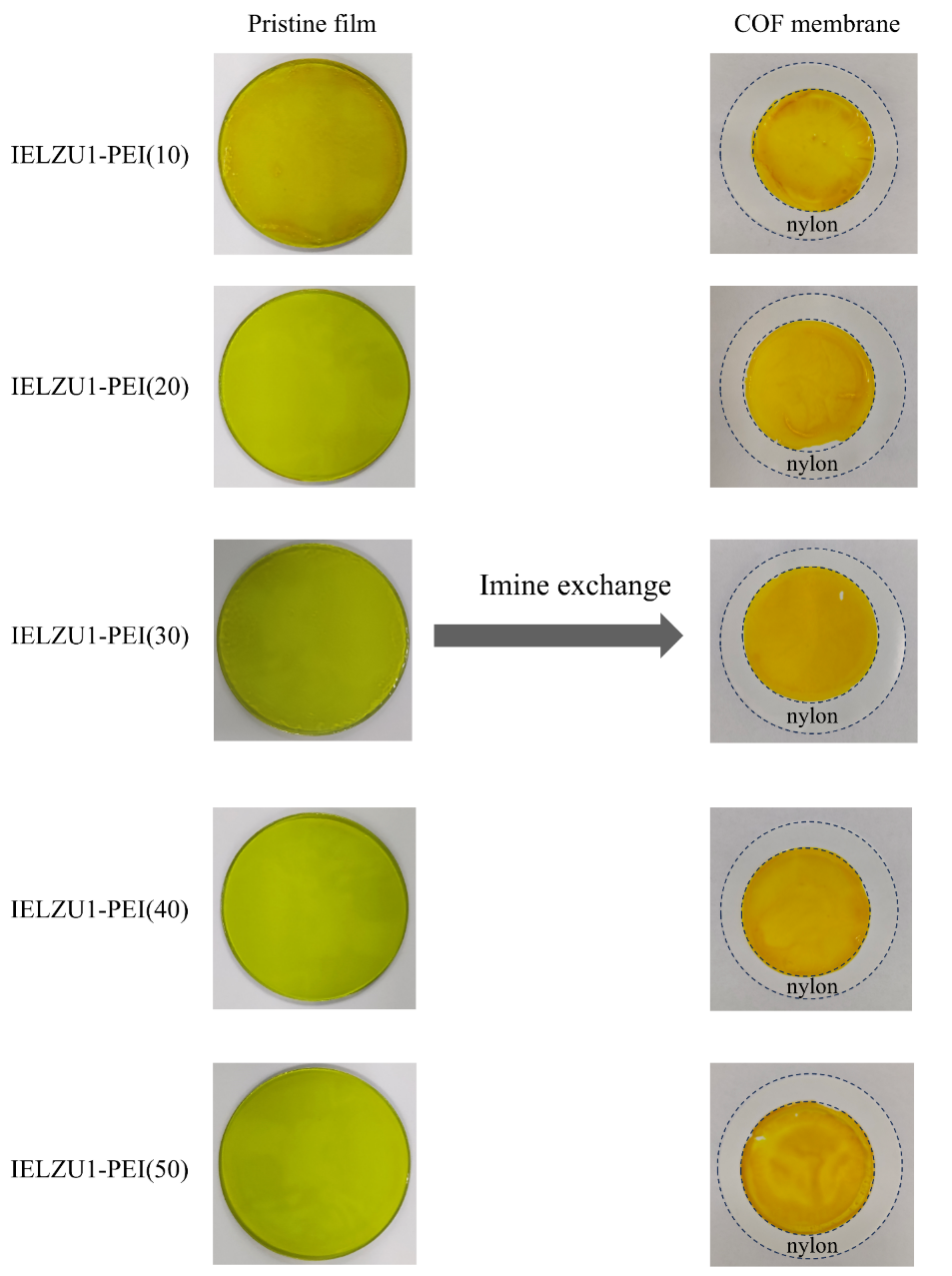


**Fig. S36** Optical images of pristine film and COF membranes at different amounts of PEI. As the amount of PEI added increased, the uniformity of the COF membrane first rose and then declined, and this trend had a strong correlation with the crystallinity of the membrane. This demonstrated that PEI amount played a crucial role in regulating the uniform distribution of crystals and the imine exchange reaction of IELZU1-PEI membrane.


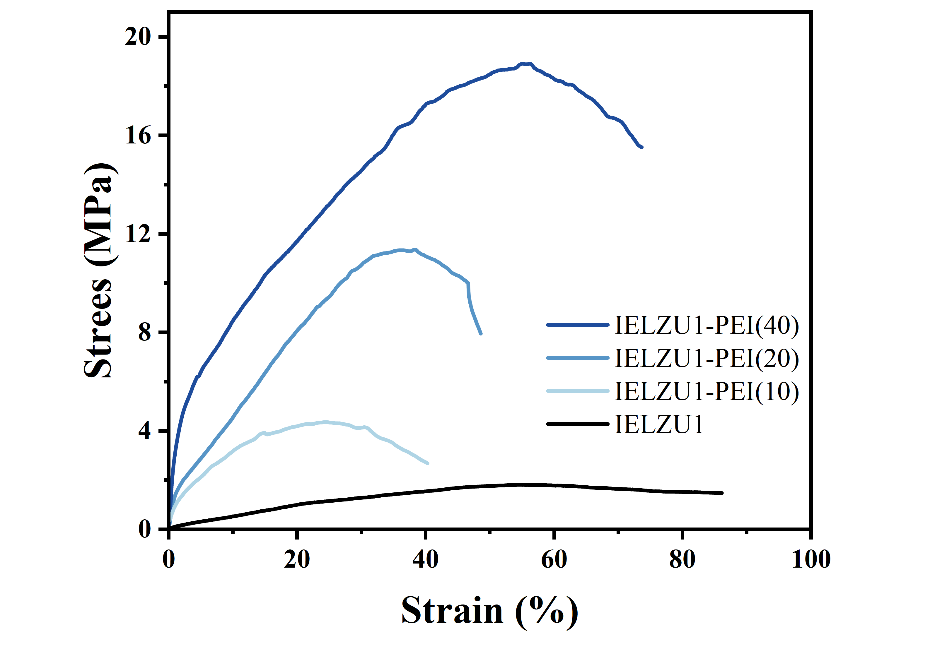


**Fig. S37** Stress-strain of the COF membranes.


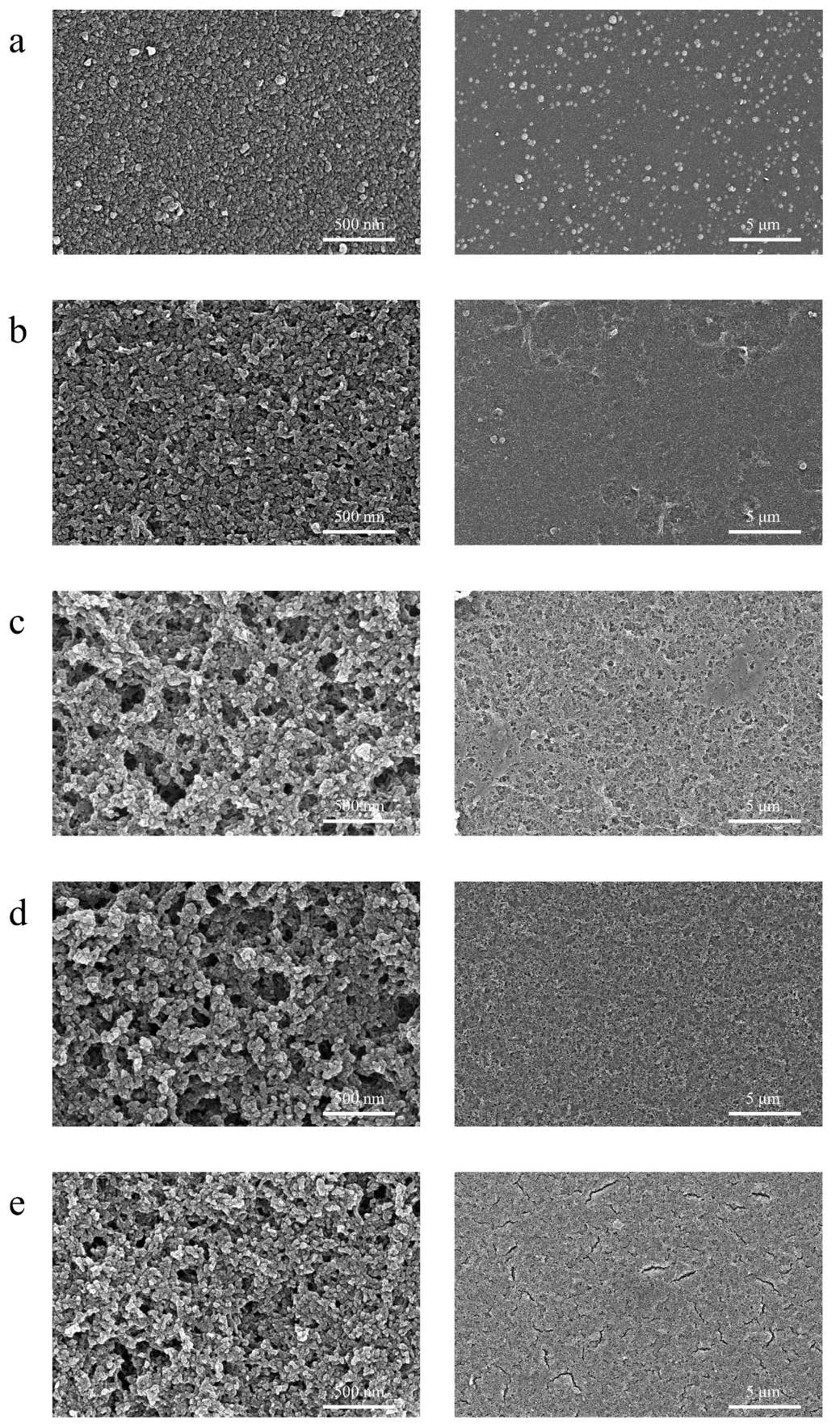


**Fig. S38 a-e** Surface SEM images of IELZU1-PEI(x) membranes at different PEI amounts: x=10 μL **a**, x=20 μL **b**, x=30 μL **c**, x=40 μL **d** and x=50 μL **e**. The above figures showed the morphology of the COF membrane close to the surface of the solvent and catalyst during imine exchange reaction.


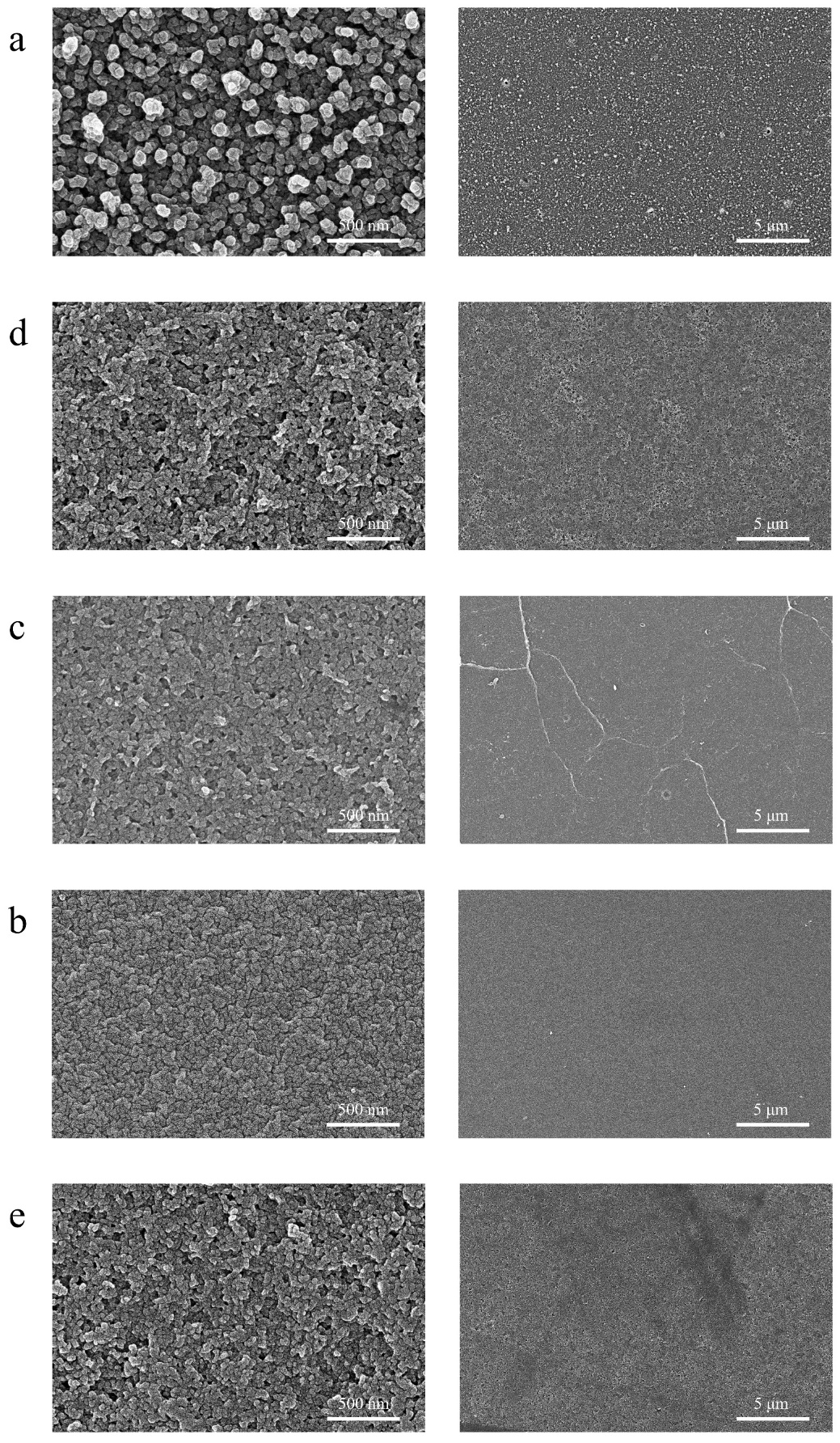


**Fig. S39 a-e** Surface SEM images of IELZU1-PEI(x) membranes at different PEI amounts: x=10 μL **a**, x=20 μL **b**, x=30 μL **c**, x=40 μL **d** and x=50 μL **e**. The above figure shows the morphology of the COF membrane close to the surface of the indium tin oxide coated plate.

**
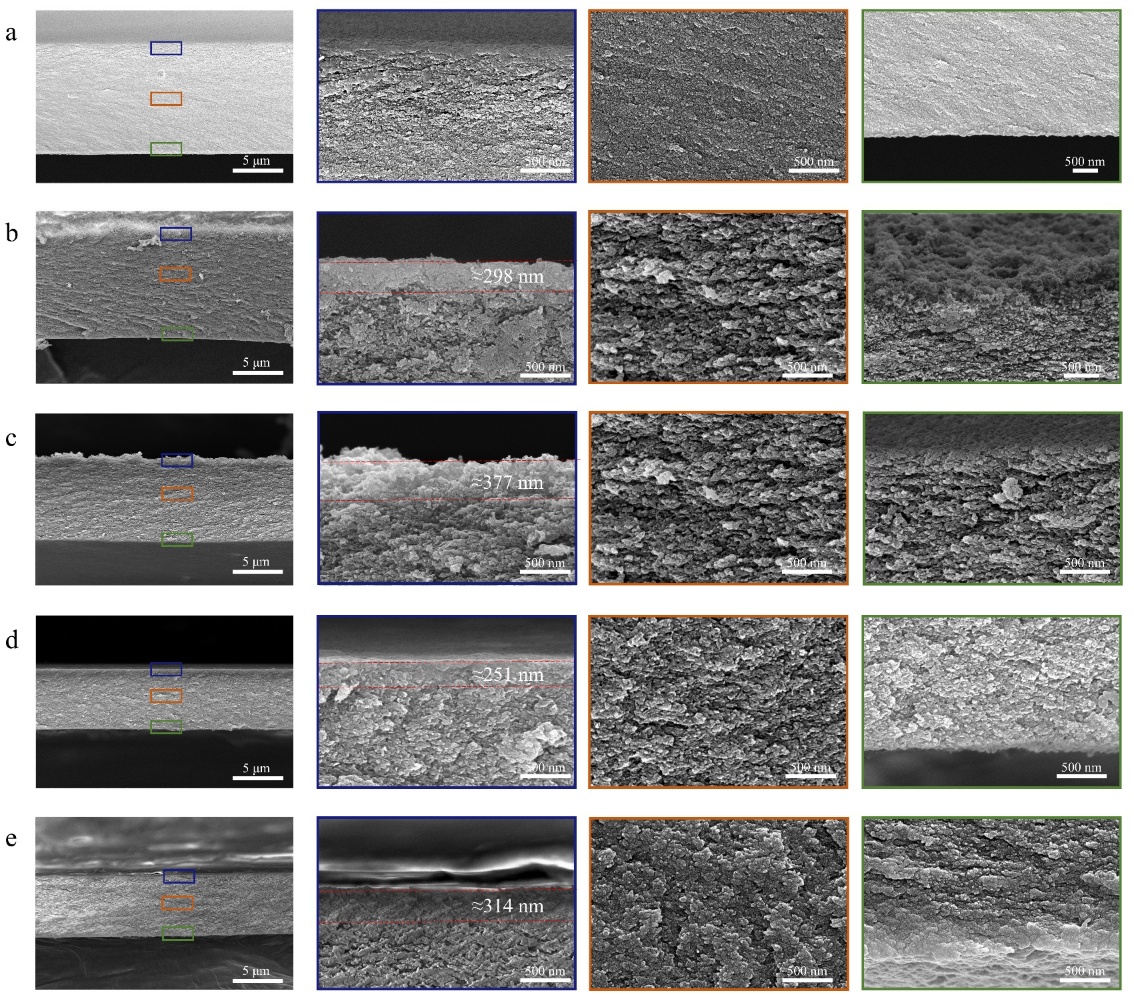
**

**Fig. S40 a-e** Cross-sectional SEM images of IELZU1 membrane **a** and IELZU1-PEI(x) membranes at different PEI amounts: x=10 μL **b**, x=20 μL **c**, x=30 μL **d** and x=40 μL **e**.


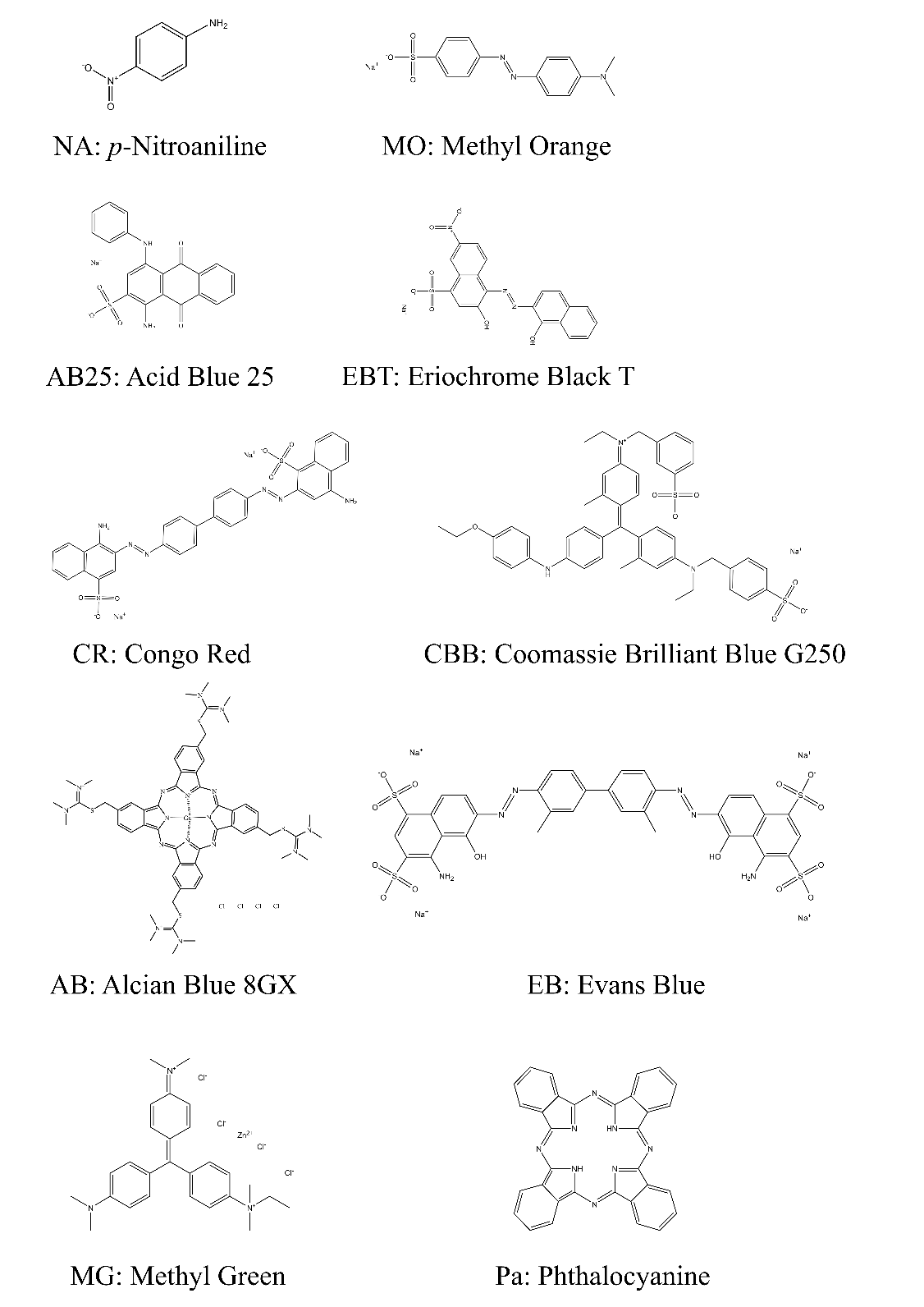


**Fig. S41** The structures of the dyes used in this work.


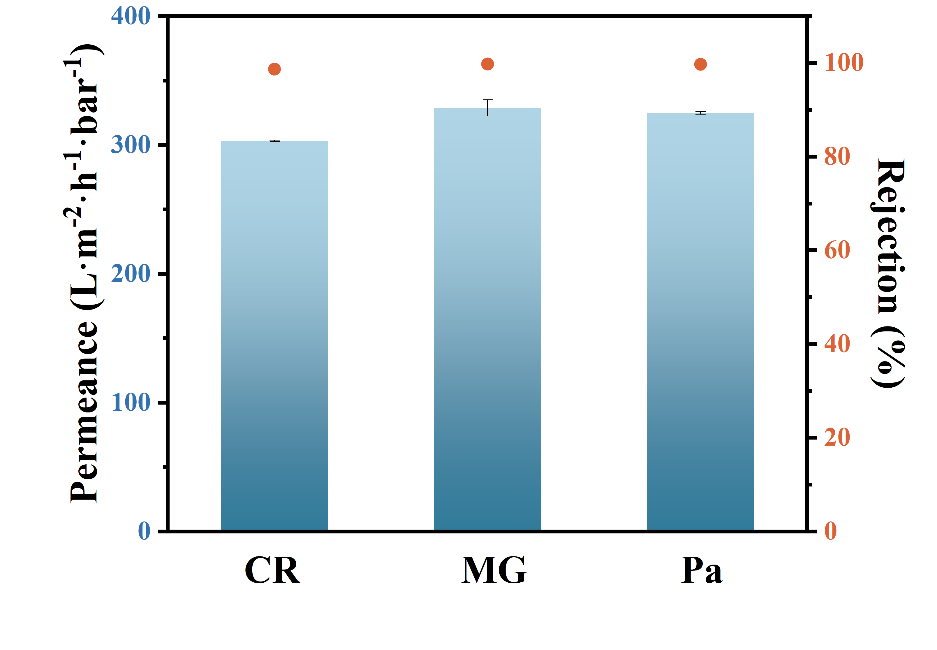


**Fig. S42** Permeance and CR rejection of IELZU1-PEI(40) membrane after saturation adsorption in different dye solutions (including CR, MG and Pa). The COF membranes were fully immersed in dye solutions (100 ppm) for 24 hours and then rinsed for water permeance and rejection testing.


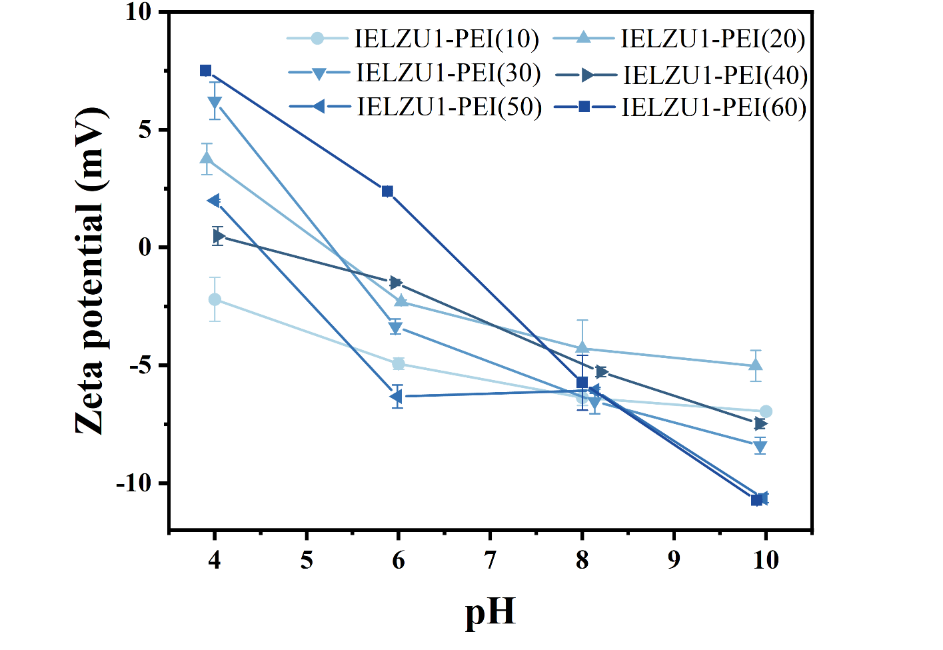


**Fig. S43** Zeta potential of IELZU1-PEI membranes.


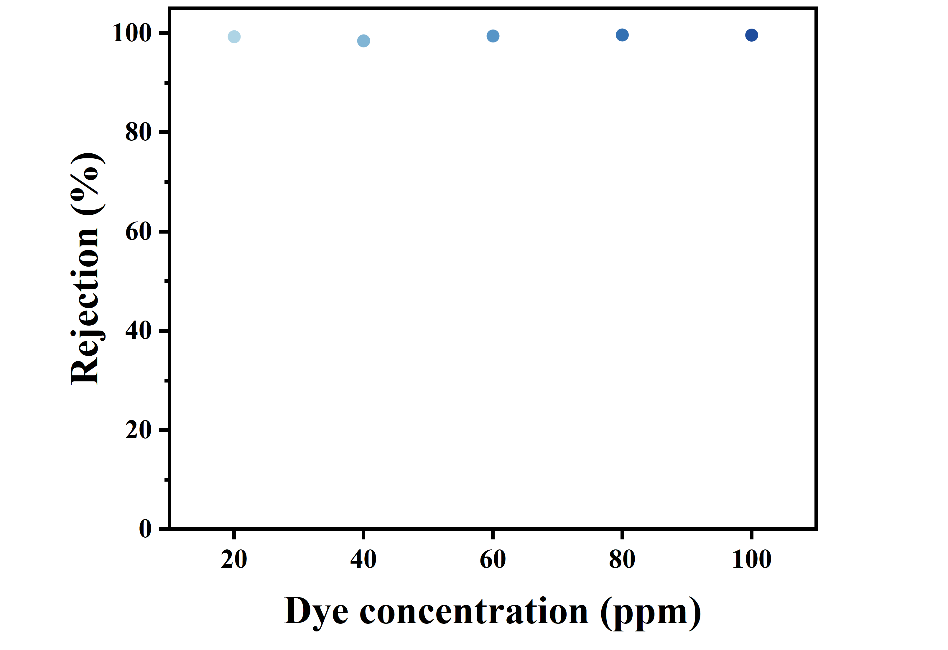


**Fig. S44** Rejection of IELZU1-PEI(40) membrane at different concentrations of CR solution.


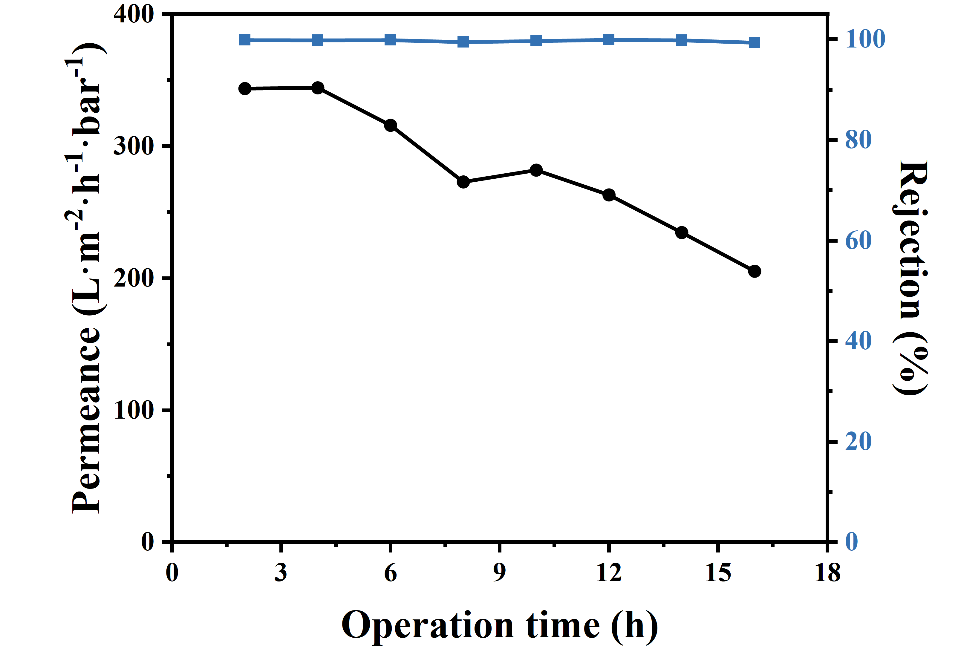


**Fig. S45** Long-term stability of IELZU1-PEI(40) membrane towards CR solution.


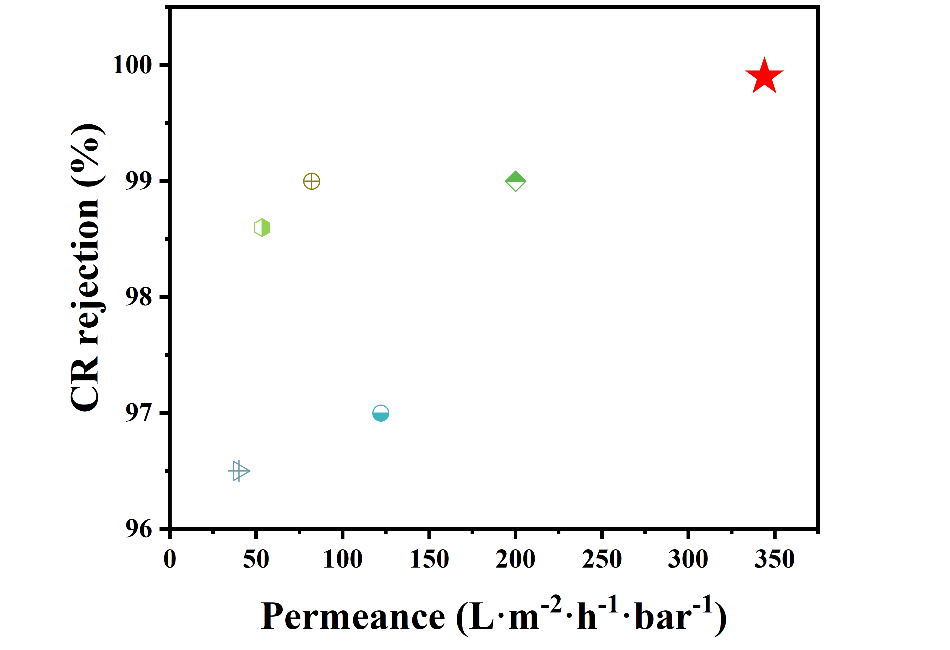


**Fig. S46** Comparison of the permeance and CR rejection IELZU1-PEI membrane with the most recently reported other LZU1 membranes.


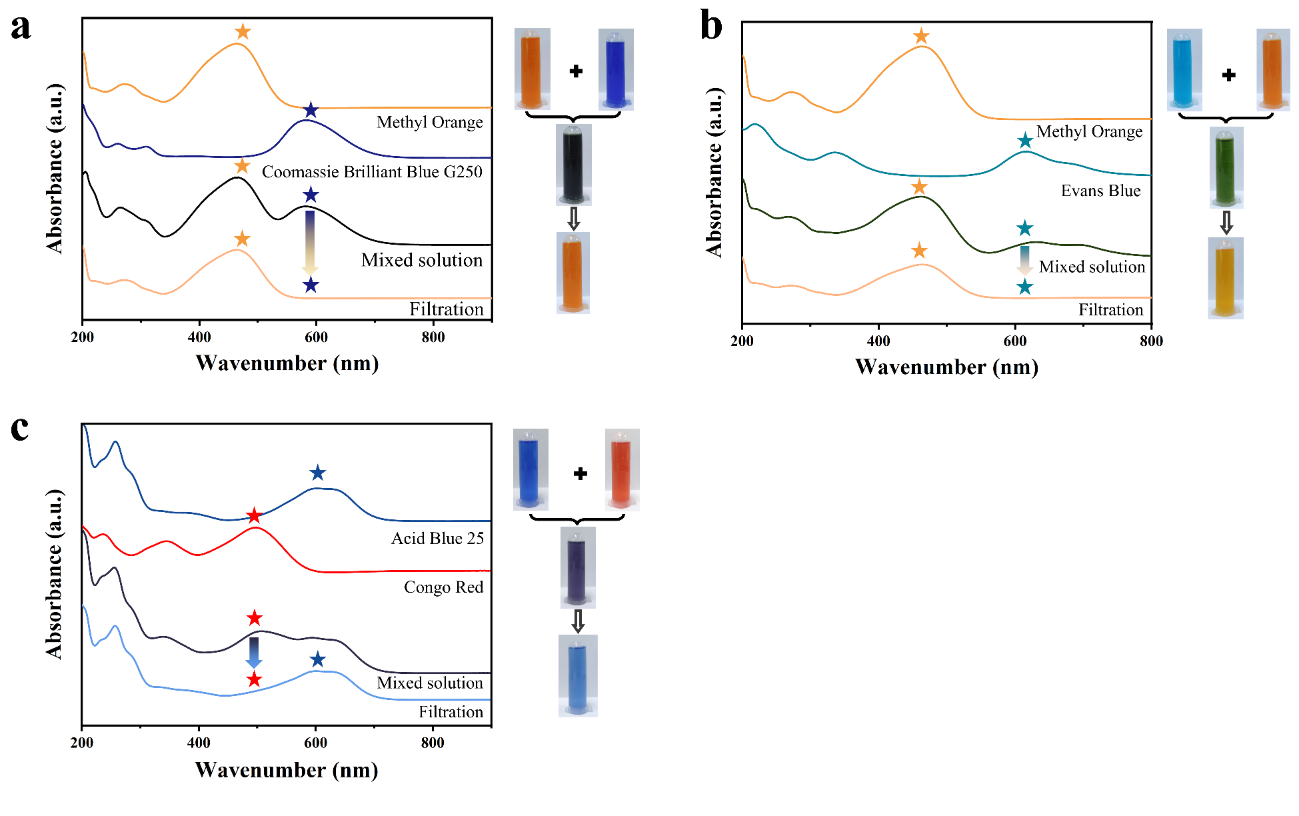


**Fig. S47 a-c** UV–vis spectra and optical images of the selective separation of the mixed dye aqueous solution of MO-CBB **a**, MO-EB **b** and AB25-CR **c**.


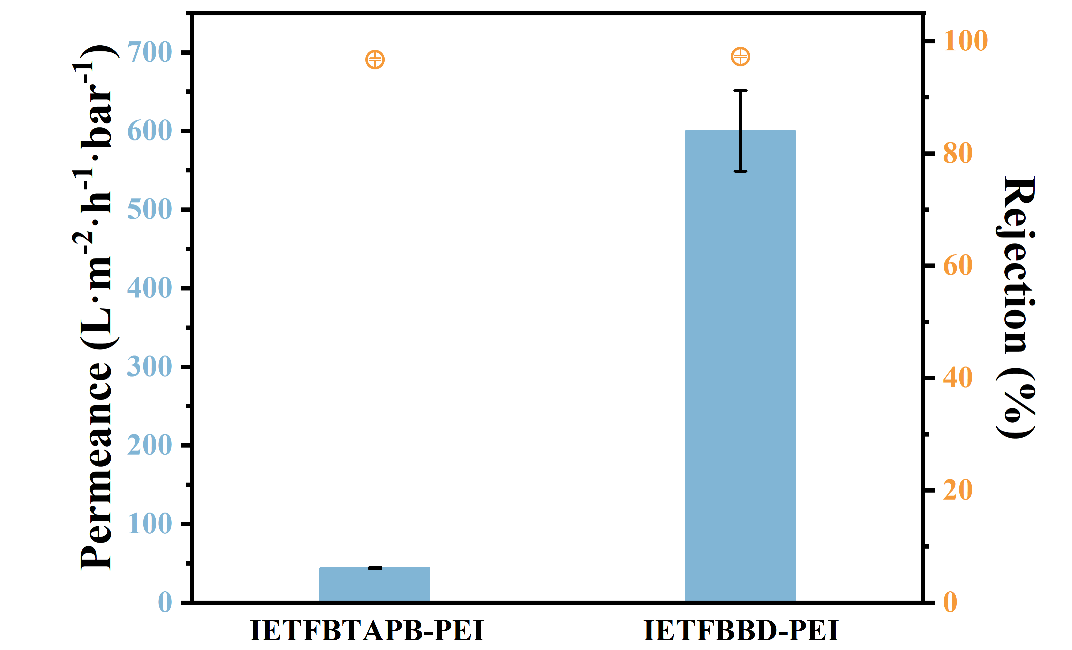


**Fig. S48** Permeance and CR rejection of IETFBTAPB-PEI membrane; Permeance and AB rejection of IETFBBD-PEI membrane. The COF membrane shown in this figure was fabricated using 40 μL of PEI.

**S3 Supplementary Tables**

**Table S1** Comparison of the nanofiltration performance, membrane fabrication methods and fabrication condition of the state-of-the-art COF membranes (take Congo Red as typical dye).

| Membrane | Dye rejection (%) | Pure water permeance  (L m^-2^ h^-1^ bar^-1^) | Fabrication  methods | Fabrication time  (h) | Fabrication temperature  (℃) | Ref. |
| --- | --- | --- | --- | --- | --- | --- |
| COF-LZU1 | 98.6 | 53.43 | Solvothermal | 75 | 120 | [S5] |
| TpPa/PVDF | 98.7 | 60 | in-situ growth | 25 | 24 | [S6] |
| TB-DA COF2 | 98.7 | 73.4 | Solvent volatilization | 0.03 | 100 | [S7] |
| TBDM | 100 | 439.4 | In-situ growth | > 24 | 25 | [S8] |
| TBDH | 86.1 | 210.3 |  | > 24 | 25 | [S8] |
| TpEB | 99.23 | 31.34 | Interface polymerization | 2 | 65 | [S9] |
| TFP-PDA | 99 | 403 | Solid-vapor  Interface  polymerization | > 18 | 60/145 | [S10] |
| TFP-TTA | 99 | 338 |  | > 18 | 60/145 | [S10] |
| TFP-PDA | 98.9 | 411 | Solid-vapor  Interface  polymerization | > 11 | 150 | [S11] |
| TpPa-SO_3_H | 92.42 | 117 | Electrophoretic Deposition | > 72 | 25 | [S12] |
| HsGDY | 99.82 | 78 | Solvothermal | > 10 | 110 | [S13] |
| TpDMTH | 99.5 | 109.73 | Interfacial catalytic polymerization | 1.5 | 60 | [S14] |
| TFG-EDA | >98 | 531 | liquid–liquid interfacial polymerization | 72 | 25 | [S15] |
| TFG-DETA | >98 | 658 |  | 72 | 25 | [S15] |
| TFG-TETA | >98 | 720 |  | 72 | 25 | [S15] |
| COFs-NH_2_PES | >99 | 429 | Interface polymerization | > 24 | 60 | [S16] |
| TpPa-SO_3_H | 99.4 | 43 | Solution casting | 48 | 60 | [S17] |
| (TG/Pa)1 | 98 | 85 | Vacuum-assisted assembly | > 125 | 25 | [S18] |
| TpPa-PVP-360/Nylon | 98 | 550 | Spray-coating | > 24 | 90 | [S19] |
| TpPa-PVP-50/Nylon | 99 | 280 |  | > 24 | 90 | [S19] |
| BNNS/ANF/COF | 98 | 194 | Vacuum-assisted assembly | > 168 | 25 | [S20] |
| TpPa–II/PMIA | 99.2 | 105.6 | In-situ growth | > 72 | 40 | [S21] |
| TA–COF–PES | >97 | 122 | Interface polymerization | > 0.5 | 60 | [S22] |

**Table S2** Comparison of the permeance and CR rejection IELZU1-PEI membrane with the most recently reported other LZU1 membranes.

| Membrane | CR rejection (%) | Pure water permeance  (L m^-2^ h^-1^ bar^-1^) | Fabrication  methods | Ref. |
| --- | --- | --- | --- | --- |
| COF-LZU1 | 98.6 | 53.43 | Solvothermal | [S5] |
| COF-TA-PES | 97.0 | 122 | Interfacial polymerization | [S22] |
| COF–LZU1 composite membranes | 96.5 | 40 | Spraying | [S23] |
| PVDF/LZU1 | 99.0 | 200 | Interfacial polymerization | [S24] |
| COF-LZU1/PES | 99.23 | 31.34 | Interface polymerization | [S25] |

**Supplementary References**

1. S. Meenakshisundaram, M. Manickam, T. Pillaiyar, Exploration of imidazole and imidazopyridine dimers as anticancer agents: Design, synthesis, and structure–activity relationship study. Archiv. Der. Pharmazie. **352**(12), 1900011 (2019). <https://doi.org/10.1002/ardp.201900011>
2. X. Wang, L. Zhang, S. He, X. Chen, X. Huang et al., Dynamic imine exchange reactions for facile synthesis of imine-linked covalent organic frameworks. Chem. Mater. **35**(23), 10070-10077 (2023). <https://doi.org/10.1021/acs.chemmater.3c02092>
3. W. Zhang, L. Chen, S. Dai, C. Zhao, C. Ma et al., Reconstructed covalent organic frameworks. Nature. **604**(7904), 72-79 (2022). <https://doi.org/10.1038/s41586-022-04443-4>
4. S.-Y. Ding, J. Gao, Q. Wang, Y. Zhang, W.-G. Song et al., Construction of covalent organic framework for catalysis: Pd/COF-LZU1 in suzuki–miyaura coupling reaction. J. Am. Chem. Soc. **133**(49), 19816-19822 (2011). <https://doi.org/10.1021/ja206846p>
5. H. Fan, J. Gu, H. Meng, A. Knebel, J. Caro, High-flux membranes based on the covalent organic framework COF-LZU1 for selective dye separation by nanofiltration. Angew. Chem. Int. Ed. **57**(15), 4083-4087 (2018). <https://doi.org/10.1002/anie.201712816>
6. R. Wang, X. Shi, Z. Zhang, A. Xiao, S.-P. Sun et al., Unidirectional diffusion synthesis of covalent organic frameworks (cofs) on polymeric substrates for dye separation. J. Membr. Sci. **586**(15), 274-280 (2019). <https://doi.org/10.1016/j.memsci.2019.05.082>
7. G. Wang, Y. Chen, C. Pan, H. Chen, S. Ding et al., Rapid synthesis of self-standing covalent organic frameworks membrane via polyethylene glycol-assisted space-confined strategy. J. Membr. Sci. **652**(15), 120494 (2022). <https://doi.org/10.1016/j.memsci.2022.120494>
8. Y. He, X. Lin, Fabricating compact covalent organic framework membranes with superior performance in dye separation. J. Membr. Sci. **637**(1), 119667 (2021). <https://doi.org/10.1016/j.memsci.2021.119667>
9. N. Basel, Q. Liu, L. Fan, Q. Wang, N. Xu et al., Surface charge enhanced synthesis of tpeb-based covalent organic framework (COF) membrane for dye separation with three typical charge properties. Sep. Purif. Technol. **303**(15), 122243 (2023). <https://doi.org/10.1016/j.seppur.2022.122243>
10. N. A. Khan, R. Zhang, X. Wang, L. Cao, C. S. Azad et al., Assembling covalent organic framework membranes via phase switching for ultrafast molecular transport. Nat. Commun. **13**(1), 3169 (2022). <https://doi.org/10.1038/s41467-022-30647-3>
11. N. A. Khan, R. Zhang, H. Wu, J. Shen, J. Yuan et al., Solid–vapor interface engineered covalent organic framework membranes for molecular separation. J. Am. Chem. Soc. **142**(31), 13450-13458 (2020). <https://doi.org/10.1021/jacs.0c04589>
12. R. Wang, Y. Zhou, Y. Zhang, J. Xue, J. Caro et al., Ultrathin covalent organic framework membranes prepared by rapid electrophoretic deposition. Adv Mater. **34**(44), e2204894 (2022). <https://doi.org/10.1002/adma.202204894>
13. X. Yang, Z. Qu, S. Li, M. Peng, C. Li et al., Ultra-fast preparation of large-area graphdiyne-based membranes via alkynylated surface-modification for nanofiltration. Angew. Chem. Int. Ed. **62**(17), e202217378 (2023). <https://doi.org/10.1002/anie.202217378>
14. J.-Y. Dai, Y.-X. Fang, Z.-L. Xu, D. Pandaya, J. Liang et al., Robust covalent organic frameworks membranes for ultrafast dye/salt separation in harsh environments. Desalination. **568**(15), 117025 (2023). <https://doi.org/10.1016/j.desal.2023.117025>
15. B. Mishra, B. P. Tripathi, Flexible covalent organic framework membranes with linear aliphatic amines for enhanced organic solvent nanofiltration. J. Mater. Chem. A. **11**(30), 16321-16333 (2023). <https://doi.org/10.1039/D3TA02683C>
16. Y. X. Liu, J. H. Chen, N. Qin, D. L. Gao, Y. Y. Zhang et al., Construction of covalent organic frameworks membranes in situ through nonsolvent-induce phase separation for fast and accurate nanofiltration. J. Membr. Sci. **697**, 122601 (2024). <https://doi.org/10.1016/j.memsci.2024.122601>
17. X. Liu, J. Wang, Y. Shang, C. T. Yavuz, N. M. Khashab, Ionic covalent organic framework-based membranes for selective and highly permeable molecular sieving. J. Am. Chem. Soc. **146**(4), 2313-2318 (2024). <https://doi.org/10.1021/jacs.3c11542>
18. X. Zhao, J. Sun, X. Cheng, Q. Qiu, G. Ma et al., Colloidal 2d covalent organic framework-tailored nanofiltration membranes for precise molecular sieving. ACS Appl. Mater. Interfaces. **15**(46), 53924-53934 (2023). <https://doi.org/10.1021/acsami.3c12106>
19. T. Ju, M. Liu, X. Shi, A. Xiao, Z. Zhang et al., Chemically asymmetric polymers manipulate the crystallization of two-dimensional covalent organic frameworks to synthesize processable nanosheets. ACS Nano. **17**(23), 23784-23793 (2023). <https://doi.org/10.1021/acsnano.3c07743>
20. D. Ning, Z. Lu, L. Hua, X. Zhang, N. Li et al., Designing nanofluidic channels of boron nitride nanosheets/aramid nanofibers/covalent organic frameworks nanofiltration membrane for ultrafast mass transport. Small. **20**(40), 2402284 (2024). <https://doi.org/10.1002/smll.202402284>
21. Q. Liu, P. Huang, N. Xu, Q. Wang, L. Fan, Decoupling synthesis and crystallization of covalent organic frameworks (cofs) for more crystalline layer targeting high efficiency dye separation. Sep. Purif. Technol. **341**(9), 126881 (2024). <https://doi.org/10.1016/j.seppur.2024.126881>
22. W. Deng, Z. Zhang, L. Liu, Z. Zhou, L. Wu, Tannin-assisted interfacial polymerization towards COF membranes for efficient dye separation. RSC Advances. **14**(23), 16510-16519 (2024). <https://doi.org/10.1039/D4RA02838D>
23. S. Hao, L. Jiang, Y. Li, Z. Jia, B. Van der Bruggen, Facile preparation of COF composite membranes for nanofiltration by stoichiometric spraying layer-by-layer self-assembly. Chem. Commun. **56**(3), 419-422 (2020). <https://doi.org/10.1039/C9CC08331F>
24. C. Wu, X. Wang, T. Zhu, P. Li, S. Xia, Covalent organic frameworks embedded membrane via acetic-acid-catalyzed interfacial polymerization for dyes separation: Enhanced permeability and selectivity. Chemosphere. **261**, 127580 (2020). <https://doi.org/10.1016/j.chemosphere.2020.127580>
25. Y.-Y. Su, X. Yan, Y. Chen, X.-J. Guo, X.-F. Chen et al., Facile fabrication of COF-LZU1/pes composite membrane via interfacial polymerization on microfiltration substrate for dye/salt separation. J. Membr. Sci. **618**(15), 118706 (2021). <https://doi.org/10.1016/j.memsci.2020.118706>
